# CLEC12A sensitizes differentially responsive breast cancer cells to the anti-cancer effects of artemisinin by repressing autophagy and inflammation

**DOI:** 10.3389/fonc.2023.1242432

**Published:** 2023-12-08

**Authors:** Ranodeep Chatterjee, Aditya Shukla, Kausiki Chakrabarti, Urmi Chatterji

**Affiliations:** ^1^ Cancer Research Laboratory, Department of Zoology, University of Calcutta, Kolkata, India; ^2^ Cell Biology Laboratory, Department of Microbiology, University of Calcutta, Kolkata, India; ^3^ Department of Zoology, Charuchandra College, Kolkata, India; ^4^ Centre for Research in Nanoscience and Nanotechnology, University of Calcutta, Kolkata, India

**Keywords:** Clec12A, artemisinin, TLR4, autophagy, apoptosis, cancer stem cells

## Abstract

**Background:**

Enhanced inflammatory responses promote tumor progression by activating toll-like receptors (TLRs), which in turn are inhibited by C-type lectin like receptors (CTLRs), like CLEC12A. Although the presence of CLEC12A in acute myeloid leukemia is well established, its role in non-hematopoietic tumors is still obscure. In hematopoietic tumors, CLEC12A mostly inhibits TLRs and modulates inflammatory responses via NF-κB signaling. In this study, the fate of tumor progression was determined by modulating CLEC12A using artemisinin (ART), a FDA-approved anti-malarial drug, known for its anti-cancer and immunomodulatory properties with minimal adverse effects on normal cells.

**Method:**

Effects of ART were primarily determined on hematological factors and primary metastatic organs, such as lungs, kidney and liver in normal and tumor-bearing BALB/c mice. Tumor-bearing mice were treated with different concentrations of ART and expressions of CLEC12A and associated downstream components were determined. CLEC12A was overexpressed in MDA-MB-231 and 4T1 cells, and the effects of ART were analyzed in the overexpressed cells. Silencing TLR4 using vivo morpholino was performed to elucidate its role in tumor progression in response to ART. Finally, CLEC12A modulation by ART was evaluated in the resident cancer stem cell (CSC) population.

**Results:**

ART did not alter physiology of normal mice, in contrast to tumor-bearing mice, where ART led to tumor regression. In addition, ART reduced expression of CLEC12A. Expectedly, TLR4 expression increased, but surprisingly, that of NF-κB (RelA) and JNK/pJNK decreased, along with reduced inflammation, reduced autophagy and increased apoptosis. All the above observations reverted on overexpression of CLEC12A in MDA-MB-231 and 4T1 cells. Inhibition of TLR4, however, indicated no change in the expressions of CLEC12A, NF-κB, or apoptotic markers. The effect of ART showed a similar trend in the CSC population as in cancer cells.

**Conclusion:**

This study, for the first time, confirmed a differential role of CLEC12A in non-hematopoietic tumor and cancer stem cells in response to ART. Subsequent interaction and modulation of CLEC12A with ART induced tumor cell death and abrogation of CSCs, confirming a more comprehensive tumor therapy with reduced risk of recurrence. Therefore, ART may be repurposed as an effective drug for cancer treatment in future.

## 1 Introduction

Tumor progression, circumventing the immune suppression, has been of interest for several years (1); and how tumor cells cope with immune cells remains a major concern. Most immune cells express pattern-recognition receptors (PRRs) to orchestrate an immune response (2). Interestingly the role of these receptors and their immunological functions have exceeded beyond immune cells. It is now known that cancer cells, even from non-hematopoietic origins, can employ PRRs, such as Toll-like receptors (TLRs), to modulate tumor progression and play regulatory roles that determine the outcome of tumor progression (3, 4). On the other hand, C-type lectin receptors (CLRs), another type of PRRs, sense molecular patterns from tumor cells, alter the inflammatory status and determine the fate of tumorigenesis (5, 6). Although the function of CLRs is well established in the immune system, information regarding their involvement in solid tumors is largely unknown.

CLEC12A (C-type lectin-like domain family 12 member A), an ITIM-containing inhibitory CLR, is expressed by granulocytes, monocytes, macrophages, and DCs (7, 8). It is considered a marker for AML blasts and plays a crucial role in carcinogenesis (9). CLEC12A has endogenous ligands in cells and can identify dead cells using a novel CLEC12A-immunoglobulin (Ig) fusion protein and reporter cells expressing CLEC12A-CD3ζ chimera (10). CLEC12A is an inhibitory PRR and is antagonistic to the activating PRRs, like TLRs. CLEC12A is eventually known to dampen inflammatory responses that are triggered by TLR-induced NF-kB activation (11). Interestingly, it has been suggested that ITIMs can promote immunological response rather than inhibit it, contrary to how CLEC12A is anticipated to operate (12). The dual nature of CLEC12A has constrained our understanding of this molecule and necessitates further investigation, specifically regarding cancer cells with non-hematopoietic ancestry, for therapeutic benefits.

According to the Human Protein Atlas database, there is a strong correlation between urothelial carcinoma and CLEC12A expression, although the function of CLEC12A in urothelial carcinoma is largely unraveled till date (13). Additionally, testis, stomach, and embryonal carcinomas exhibit substantial cytoplasmic staining of CLEC12A. The database indicates that 1 in 10 patients with urothelial cancer have moderate to elevated CLEC12A expression, in contrast to 1 in 12 patients with stomach and testis cancer (13). Another study showed that when CLEC12A was silenced by siRNA, LCIII expression was significantly reduced in HeLa cells, confirming that CLEC12A is involved in autophagy (14). Since till date there is no study elucidating the involvement of CLEC12A in solid tumors, we have attempted to investigate the role of CLEC12A in breast cancer cells, using a mice model.

In addition, it is well established that conventional chemotherapy mostly eliminates the rapidly proliferating tumor cells leaving behind the chemoresistant cancer stem cells (CSCs). Such remnant CSCs can lead to tumor recurrences at regional or distant sites (15), since they are difficult to target because of their relatively quiescent and drug-resistant nature (16). Therefore, chemotherapeutic drugs that can specifically target the CSCs, in addition to the bulk tumor cells, would be an effective strategy in the comprehensive treatment of cancer. Artemisinin (ART), an US Food and Drug Administration (FDA)-approved drug, used to cure fevers and chills and later malaria (17), was repurposed to treat breast tumors in mice. Artemisinin (ART) exhibits beneficial effects including antiviral and fungicidal properties, anti-inflammatory and anti-asthma effects, as well as, potential anticancer functions (18), by generating reactive oxygen species (ROS; 19). Interestingly, ART exerts minimal adverse effects on normal cells, and is therefore advantageous in reducing the risk of serious side effects post-chemotherapy (20). In this study, we have established that modifying the expression of CLEC12A in the breast tumor cells using ART can have beneficial functional consequences and translatable therapeutic approaches in future.

## 2 Materials and methods

### 2.1 Reagents

Artemisinin (ART), MTT kit, ampicillin were obtained from Sigma Aldrich, (USA). Hematology reagents were purchased from StanBio Reagents (India). Kits for serum alanine aminotransferase (ALT) and aspartate aminotransferase (AST) were obtained from Robonik (India), creatinine and urea from Coral Clinical Systems (India). For histology, paraffin was obtained from Leica, Germany and Hematoxylin-Eosin stains were purchased from SRL Chemicals, India. Collagenase-hyaluronidase cocktail from Stem Cell Technologies (Canada) and 3-(4,4-dimethylthiazol-2-yl)-2,5-diphenyltetrazolium bromide (MTT) solution from Sigma Aldrich (USA). Propidium Iodide-FITC Annexin Apoptosis detection kit was acquired from Bio Legend (USA), acridine orange and DCFDA from Abcam (UK). Multianalyte ELISArray kit (Cat. No. 008A) was from Qiagen (Hilden, Germany), Aldefluor™ reagents were from Stem Cell Technologies (USA). All antibodies were obtained from Santa Cruz Biotechnologies (USA). Primers for CLEC12A were procured from Integrated DNA Technologies, USA., T4 DNA ligase, BamHI and EcoRI (New England BioLabs; USA), Lipofectamine^®^ 2000 reagent (Invitrogen, USA) and pcDNA3.1(+) was a kind gift from Cell Biology Lab, Department of Microbiology, University of Calcutta. Vivo-morpholino oligos for TLR4 was purchased from GeneTools LLC, USA.

### 2.2 Cell culture

Human triple negative breast cancer cells MDA-MB-231 and murine mammary cancer cells 4T1 were obtained from National Centre for Cell Sciences, Pune, India. Cells were maintained in complete Dulbecco’s Modified Eagle Medium (DMEM) supplemented with 10% FBS and 1% penicillin/streptomycin, and incubated at 37°C in 5% CO_2_ - 95% humidified atmosphere. Cells in log phase of growth was used for all subsequent experiments (21).

### 2.3 Animals

Female BALB/c mice, weighing 20g ± 2g were purchased from West Bengal Live Stock Development Corporation, Government of West Bengal, India. Animals were housed under standard laboratory conditions. 50% ± 10% relative humidity, 20°C ± 2°C temperature and normal 12h light/dark cycle were maintained throughout the experiment with food and water ad libitum. The animals were allowed to acclimatize for 7 days prior to commencement of experiments (22). All procedures involving animals were in accordance with the Principles of Laboratory Animal Care (NIH Publication No 85-23, revised in 1985) and approval of the Institutional Animal Ethical Committee, Government of India (Registration Number 885/ac/05/CPCSEA). Specific Indian Laws of Animal Protection (ILAP) were followed throughout the experimental schedule.

### 2.4 Tumor development and treatment with artemisinin

Mouse mammary 4T1 cells in passage numbers between 7 and 12 (10^4^ cells/ml in 50 µl PBS) were injected into the inguinal 4th mammary fat pad of BALB/c mice (23, 24). Normal mice received an equal volume of sterile PBS. A palpable tumor was allowed to develop for 14 days (24). Following tumor development, mice were randomly divided into 6 groups (n=5) and treated intraperitoneally with different doses of artemisinin for 7 days, as follows: Group I - Normal (Tumor-free BALB/c mice, received the vehicle); Group II - Tumor (Mammary tumor-bearing mice, received the vehicle); Group III - T + 6.25 (Tumor-bearing mice, received 6.25 mg/kg body weight of ART); Group IV - T + 12.5 (Tumor-bearing mice, received 12.5 mg/kg body weight of ART); Group V - T + 25 (Tumor-bearing mice, received 25 mg/kg body weight of ART); and Group VI - T + 50 (Tumor-bearing mice, received 50 mg/kg body weight of ART). Normal (mammary fat pad surrounding the 4^th^ inguinal mammary pad), mammary tumor, and organs like lungs, kidney, liver, and blood were collected at the end of the experimental schedule. Body weight and tumor volume of each mouse were recorded daily during the tenure of the experiment. Tumor volume was calculated using the following formula: length (mm) × width^2^ (mm) × 0.52 (25).

### 2.5 Mean survival time

To evaluate the mean survival time of tumor-bearing mice with or without ART treatment, mortality was recorded for each group (n=5) over a period of 90 days. MST was calculated using the equation: MST (days) = (day of first mortality + day of last mortality)/2. The increase in survivability between treated and untreated groups was estimated by ILS% = [(MST of treated set/MST of untreated set) -1] X 100 (26).

### 2.6 Hematology

Blood was drawn for hematological estimations by thoracotomy and collected in heparinized vials. Blood was diluted using RBC diluting fluid (1: 1000) and WBC diluting fluid (1:100), and RBCs and WBCs were counted using a Neubauer counting chamber. Differential count was performed on blood smear stained with Leishman stain and observed under a microscope at 40X magnification (Dewinter Optical Inc., India). The percentage of lymphocytes, neutrophils, monocytes, eosinophils and basophils were recorded according to their nuclear structure (27). Hemoglobin content was estimated using Drabkin’s solution (1g sodium bicarbonate, 0.05g potassium cyanide and 0.2g potassium ferricyanide per liter), which lysed RBCs to convert hemoglobin to cyanmethemoglobin. Finally, the hemoglobin content of the test and the standard samples were obtained in gm/dl by recording the O.D. at 540 nm, and using the formula: O.D of test/O.D of standard x concentration of standard (28).

### 2.7 Serum biochemical parameters

Levels of alanine aminotransferase (ALT) and aspartate aminotransferase (AST) from the serum were determined using ALT and AST estimation kits (Robonik India Pvt. Ltd, India). Briefly, for ALT activity estimation, Reagent 1 (Tris Buffer, L-Alanine, Lactate Dehydrogenase) and Reagent 2 (α-ketoglutarate, NADH) was mixed (4:1) with 100 µl serum. After 60 seconds incubation, absorbance was measured at 340 nm every minute for 180 seconds (ΔA/minute). Finally, the activity was estimated using the formula: Activity of the sample = (ΔA/minute x 1746). Analysis and final calculations were carried out according to the manufacturer’s protocol (29) For AST activity estimation, Reagent 1 (Tris Buffer, L-Aspartate, Lactate Dehydrogenase; >600U/L, Malate dehydrogenase; >600U/L) and Reagent 2 (α-ketoglutarate, NADH) was mixed together (4:1) with 100 µl serum. After incubation for 60 seconds, absorbance was measured at 340 nm per minute for 180 seconds (ΔA/minute). Finally, the activity was estimated using the formula: Activity of the sample = (ΔA/minute x 1746). Analysis and final calculations were carried out according to the manufacturer’s protocol (30). For estimation of creatinine level, picric acid (0.5 ml), buffer (0.5 ml) and sample (Test)/standard (S) (0.1 ml) were mixed and absorbance was measured at 520 nm for 30 seconds and 60 seconds. The rate of change of absorbance was determined for the standard (ΔS) and test (ΔT) samples. The levels were estimated using the formula: creatinine (mg/dl) = (ΔT)/(ΔS) x 2.0 (31). Urea levels were estimated using specific metabolite analysis kits. Buffer (1 ml), enzyme (0.1 ml), sample (test)/standard (0.1 ml) and chromogen reagent (0.2 ml) were mixed. Absorbance was measured at 570 nm after 5 minutes of incubation of test (Abs T) and standard (Abs S) samples. The levels were estimated using the formula: Urea (mg/dl) = (AbsT)/(AbsS) x 40. Analysis and calculations were carried out according to the manufacturer’s protocol (32).

### 2.8 Histology

Mice were euthanized after treatment; tissues were collected and fixed in Bouin’s solution. After fixation, tissues were dehydrated in gradient ethanol and embedded in paraffin blocks. For histological studies, 5 µm tissue sections were prepared using a rotatory microtome (Leica, USA) and transferred onto glass slides. The tissues were stained using the hematoxylin-eosin double staining procedure (33). In brief, the slides were deparaffinized with xylene and. The sections were then stained with hematoxylin followed by eosin, dehydrated, mounted and documented under a light microscope (Olympus BX53; 40X magnification). The stained slides were observed and multi-nucleated cells indicative of mitotic tumor cells were counted. Counting started from the most densely accumulated areas which had the highest number of multi-nucleated cells. The next field was selected randomly, to eliminate higher counts from dense areas alone. Four sets of 25 microscopic fields (100 fields) were counted. The total number of multi-nucleated cells counted per 100 fields were represented as percent of these cells per section (34).

### 2.9 Isolation of cells from tissue

Tissues (mammary fat pad and tumors) were collected from normal and tumor-bearing mice with or without ART treatment, and were subjected to single cell digestion using the collagenase-hyaluronidase cocktail enzyme (3000U/mL collagenase, 1000U/mL hyaluronidase) in sterile DMEM. Tissue samples were immersed in 9 parts of serum-free DMEM and 1 part of enzyme cocktail. Digestion was performed at 37°C for 4 hours. Isolated cells were washed well to remove any remaining enzyme, resuspended in ice-cold PBS and filtered through a single cell mesh to dissociate cell clumps. Cell viability was checked using trypan blue dye exclusion test before using the cells for further assays (21).

### 2.10 Cell viability assay

1 x 10^5^ viable cells were seeded in a 96-well plate. Cells were treated with different concentrations of ART for 24 hours. Following treatment, wells were washed to remove the media and 100 µl of PBS was added to each well. Finally, 10 µl of MTT solution (5 mg/mL in PBS) was added and incubated at 37°C for 3 hours. The purple crystals formed were dissolved in 150 µl of DMSO. Absorbance was measured at 570 nm in a multi-plate reader Varioskan™ Lux (Thermo Fisher Scientific, USA; 21).

### 2.11 Cell cycle assay

Cells (1 x 10^6^) were isolated from tissues by collagenase-hyaluronidase digestion and fixed in ice-cold ethanol at -20°C. Samples were washed and resuspended in PBS containing 40 µg/mL RNase A for 1 hr. The cells were then stained with 30 µg/mL propidium iodide in the dark for 30 minutes and analyzed by flow cytometry using the BD Verse (BD Biosciences, USA). Data were analyzed using the BD FACSuite™ software (35).

### 2.12 Estimation of reactive oxygen species

Cells were isolated from treated normal and treated and untreated tumor tissues by collagenase-hyaluronidase digestion, Intracellular ROS was determined in cells using an oxidation sensitive fluorescence probe DCFDA. In the presence of intra-cellular esterase DCFDA is deacetylated to 2’, 7’- dichlorofluorescein (DCF). 20 µM DCFDA was added to 10^6^ viable cells and incubated for 30 minutes at 37°C. The cells were then analyzed using BD Accuri C6 (BD Biosciences, USA; 36).

### 2.13 ALDH^+^ population estimation assay

Mammary fat pad and tumor tissues (1 × 10^6^ cells/ml) were resuspended in aldefluor™ assay buffer containing ALDH substrate and incubated for 45 minutes at 37°C. As a control, cells were resuspended in buffer containing aldefluor substrate in the presence of diethylaminobenzaldehyde (DEAB), a specific ALDH inhibitor. The fluorescent ALDH^+^ cells were detected in the green fluorescence channel (520-540nm) in a flow cytometer (BD FACS Aria III). ALDH^+^ and ALDH^-^ cells were eventually sorted using the BD Aria III Cell Sorter and sorted cells collected for further analysis (BD Biosciences, USA; 21).

### 2.14 Estimation of autophagy

Acidic vesicular organelles (AVOs) were examined by microscopy and quantified using flow cytometry of acridine orange (AO) stained cells. Cells were isolated from tissues by collagenase-hyaluronidase digestion and stained with AO (1µg/ml) by incubation at 37°C for 15 minutes in dark. Following subsequent PBS washes, the cells were observed under Olympus laser-scanning confocal microscope (FV-10 ASW 3.0 viewer image browser) at 450 and 593 nm to determine AVOs. AO stained cells were also analyzed in a flow cytometer (red channel, 630 nm) using the BD Accuri (BD Biosciences; USA) to estimate the percentage of cells undergoing autophagy following ART treatment (36).

### 2.15 Analysis of apoptotic cells

Cells, isolated from normal and treated/untreated tumor tissues by collagenase-hyaluronidase digestion, were washed with Ca^2+^-free PBS and stained with Annexin V binding buffer (1X). The cells were then incubated with Annexin V-FITC and propidium iodide (PI) for 15 minutes at 37°C. Cells were eventually analyzed on the BD FACS Aria III (Alexa Fluor at 488nm and PE at 570 nm, respectively). The percentage of apoptotic and necrotic cells were analyzed using the FACSDIVA™ software (BD Biosciences, USA; 37).

### 2.16 Western blot analysis

Normal and tumor tissues were homogenized in ice-cold radioimmunoprecipitation assay (RIPA) buffer containing a protease inhibitor cocktail. Proteins were resolved by 10% sodium dodecyl sulphate-polyacrylamide gel electrophoresis (SDS-PAGE), transferred onto polyvinylidene difluoride (PVDF) membranes, blocked with 5% fat-free milk and incubated with primary antibodies (1:1000 dilution) overnight at 4°C. Blots were subsequently washed and incubated with respective secondary antibodies, tagged with horseradish peroxidase. Protein expression was detected using enhanced chemiluminescence (ECL) kit (37). Blots were analyzed using the Gel Doc XR and band intensity quantified using ImageJ software (NIH, USA).

### 2.17 Cytokine analysis

Serum isolated from experimental mice, and conditioned media collected from MDA-MB-231 and 4T1 cells, were used to estimate levels of cytokines by ELISA. To prepare the conditioned media, the cell culture medium containing serum was discarded and cells were washed with serum-free DMEM media. This was followed by incubation of the cells in serum-free media so that major proteins are not masked for further analysis (38), along with ART for 16h. After incubation, the conditioned media was collected for cytokine analysis. As per the manufacturer’s protocol, samples were added to the multi-analyte ELISArray wells, coated with antibodies for TNF-α, IL-1β, IL-6 and IL-12 and incubated for 2 hours. Detection antibodies were added, followed by 30 minutes incubation with avidin-HRP. After incubating with the developing solution, stop solution (Qiagen) was added to terminate the reaction (39). The O.D. was measured at 450 and 570 nm in an ELISA plate reader (Varioskan Lux, Thermo Fisher; USA).

### 2.18 Construction of CLEC12A-pcDNA3.1(+)

The pcDNA3.1(+) expression vector was used to overexpress CLEC12A in cancer cells. Genomic DNA (gDNA) was isolated from MDA-MB-231 and 4T1 cells using phenol chloroform (40). Forward and reverse primers were designed from the full length CLEC12A gene (NCBI, Gene ID: 160364) and restriction sites for BamHI and EcoRI (Forward: 5’-CGCGGATCCCTATTTAGCATTGCTGCTGC-3’ and Reverse: 5’-CCGGAATTCTTGCCTTTTCGTTTTTGTT-3’), were incorporated respectively. The CLEC12A gene was amplified from the gDNA template in a thermal cycler (Applied Biosystems, USA) for 35 cycles by polymerase chain reaction (PCR) (22). The PCR product and pcDNA3.1(+) expression vector were double digested with BamHI and EcoRI, and the digested fragments were ligated using T4 DNA ligase overnight at 16°C. The ligated products were transformed into E. coli DH5α competent cells, and grown in Luria-Bertani broth with 100 µg/ml ampicillin (41). Plasmids were isolated from transformed bacterial colonies and the presence of CLEC12A gene was confirmed by colony PCR assay. pcDNA3.1(+) with CLEC12A gene was designated as pcDNA-CLEC12A.

### 2.19 Cell transfection

For transfection, MDA-MB-231 and 4T1 cells were grown in a 6-well plate until they reached 70% confluency. CLEC12A-pcDNA recombinant/empty DNA plasmid (2500ng) or pcDNA empty vector (2500ng) was transfected into cells using 5μl Lipofectamine^®^ 2000 reagent. After 24 hours, transfected cells were treated without or with ART for another 24 hours. Finally, transfection efficiency was confirmed by western blot analysis (42).

### 2.20 Vivo-morpholino treatment

Murine mammary tumors were allowed to develop in BALB/c mice injected with 10^7^ 4T1 cells. Tumor-bearing mice were injected intravenously with vivo-morpholino for TLR4 (VMO-TLR4; 5’ CAGGGAGGCATCATCCTGGCATTTT 3’) or scramble morpholino (SC-MO) every other day for a week (n=3), along with ART (25 mg/kg) for 7 consecutive days (43). Control groups were set up, where normal mice and mice-bearing tumors received PBS for 7 days. At the end of the experiment, tissues were harvested and processed for subsequent experiments.

### 2.21 Molecular docking

Docking studies were performed with AutoDock 4.2.6 using the pdb file of CLEC12A from Swiss Model Repository (SMR; UniProtkB ID: Q5QGZ9). The structure of ART (sdf file) was obtained from the ChEBI database (CHEBI: 223316). The software AutoDock Tools 1.5.6 was utilized to eliminate the water molecules, isolate the proteins, add Kollman charges and nonpolar hydrogen, and saved as PDBQT format (44). The sdf format of ART was converted to a pdb file using Open Babel ver 2.3.1 (45). Torsion root was detected for ART and saved as PDBQT format using AutodockTools. A 3D grid box was considered based on the ligand-binding surface of CLEC12A on InterPro database using UniprotKB ID: Q5QGZ9 to generate grid maps. Ten docking runs were carried out using the Lamarkian Genetic algorithm and the best docking result was selected for further analysis. Molecular graphics and analyses were performed using AutoDock Tools.

### 2.22 Statistical analysis

All the data in this study was analyzed by GraphPad Prism 5.0 (GraphPad Software Inc., USA) and expressed as mean ± SD of three independent experiments. Significance between two groups was carried out by Students’ *t*-test. P values < 0.05 were considered to be statistically significant. Cell population in flow cytometry analyses was represented graphically after quantification using relevant software.

## 3 Results

### 3.1 Artemisinin reverted the altered physiological parameters of tumor-bearing mice without affecting the physiology of normal mice

**Figure 1:**
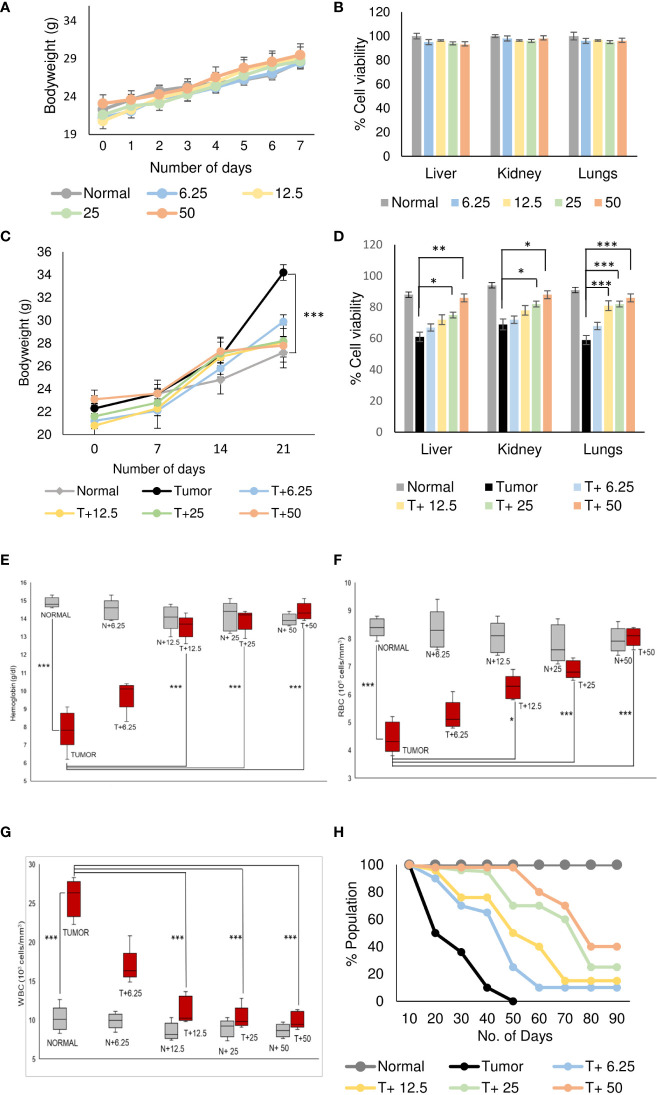
Artemisinin specifically resurrects physiological homeostasis in mice with tumors. **(A)**, Healthy mice were treated with different doses (6.25, 12.5, 25 and 50 mg/kg body weight) of ART for 7 days. The body weight of ART-treated mice indicated regular increase as observed in normal mice. **(B)**, The percentage of viable cells indicated no loss of viability of cells isolated from liver, kidney, and lungs of normal mice, in response to varying doses of ART treatment. **(C)**, Body weight of tumor-bearing mice indicated significant (p<0.001) increase compared to mice without tumors, 21 days after orthotopic implantation of 4T1 cells. In contrast, treatment with ART for 7 days (days 15 to 21) significantly reversed the body weight to that observed in normal mice. **(D)**, Cell viability of liver, kidney, and lung tissues, which radically reduced in tumor-bearing mice, increased in a dose-dependent manner when tumor-bearing mice were exposed to increasing concentrations of ART. **(E–G)**, Comparison of hemoglobin content **(E)**, RBC **(F)** and WBC **(G)** counts in tumor-bearing mice in response to ART treatment. There was a significant reduction in the hemoglobin content and RBC count (p<0.001) in mice bearing tumors compared to healthy mice. Concomitant increase in WBC count (p<0.001) was observed in tumor-bearing mice. Interestingly, all aberrations in hematological parameters were significantly restored towards normal values in a dose-dependent manner in mice treated with ART. **(H)**, Kaplan-Meier survival plot of healthy mice, mice with tumors and tumor-bearing mice treated with varying doses of ART (T+6.25, T+12.5, T+25, T+50), observed for a period of 90 days. Healthy mice survived beyond 90 days whereas mice bearing tumors succumbed within 50 days. Survivability of tumor-bearing mice improved considerably on ART treatment. Data are presented as mean ± SD and are representative of three independent experiments. ^*^p<0.05; ^**^p<0.01; ^***^p<0.001; n=5.

Normal BALB/c mice (n=5) were treated with different doses of ART, selected based on various literature (46–48) and preliminary experiments in the lab. ART treatment for 7 days did not alter the body weight of normal mice compared to vehicle treatment (**Figure 1A**). As assessed by the MTT assay, there was no loss of viability of cells isolated from the liver, kidney and lungs of ART-treated mice, confirming no apparent toxicity to these organs upon ART treatment (**Figure 1B**). Thereafter, the efficacy of ART was tested in tumor-bearing mice. It was observed that compared to normal mice, there was an increase in body weight (p<0.001) of tumor-bearing mice. However, on ART treatment, body weight of mice was restored towards normal values in a dose-dependent manner (**Figure 1C**). In addition, cells isolated from the liver, kidney and lungs of tumor-bearing mice showed significant loss of cell viability (**Figure 1D**), and severe histo-architectural damage to these organs, in addition to increased expression of the apoptosis marker, cleaved PARP (**Supplementary Figure 1**). However, increasing doses of ART significantly increased cell viability in these organs in a dose-dependent manner (**Figure 1D**), and higher doses reverted viability to levels observed in normal mice. In addition, hepatic dysfunction was assessed by serum levels of alanine transaminase (ALT) and aspartate transaminase (AST) enzymes, and renal dysfunction by the serum levels of creatinine and urea. ART treatment for 7 days did not indicate any apparent discrepancies in normal mice (**Table 1**). Preliminary studies interestingly indicated that ART treatment in normal mice for a period of three weeks also did not show any significant alterations in serum ALT, AST, creatinine and urea levels (**Supplementary Table 1**). Untreated tumor-bearing mice, on the other hand, showed anomalies in serum biochemical parameters, but upon ART treatment, these levels normalized in tumor-bearing mice (**Table 2**).

**Table 1:** Biochemical parameters of normal mice treated with ART for 7 days.

ART (mg/kg body wt)	ALT (U/l)	AST (U/l)	Creatinine (mg/dl)	Urea (mg/dl)
Normal	28.3 ± 1.6	33.8 ± 1.1	0.65 ± 0.11	27.1 ± 0.5
6.25	23.7 ± 1.8	27.3 ± 0.8	0.51 ± 0.09	24.5 ± 1.2
12.5	26.6 ± 2.2	29.6 ± 1.3	0.77 ± 0.13	26.3 ± 1.6
25	30.7 ± 1.5	31.7 ± 3.1	0.62 ± 0.07	28.2 ± 0.8
50	26.4 ± 2.3	29.3 ± 2.6	0.71 ± 0.1	27.1 ± 1.3

Serum levels of ALT, AST, creatinine and urea were assessed. Normal mice were treated with the vehicle only. No significant changes were noted in mice treated with varying doses of ART in comparison to untreated normal mice (n=5). Each analysis was performed three times. ART - artemisinin; ALT - alanine aminotransferase; AST - aspartate aminotransferase.

**Table 2:** Artemisinin restores serum levels of ALT, AST, creatinine and urea in tumor-bearing mice.

ART (mg/kg body wt)	ALT (U/l)	AST (U/l)	Creatinine (mg/dl)	Urea (mg/dl)
Normal	28.3 ± 1.6	33.8 ± 1.1	0.65 ± 0.11	27.1 ± 0.5
Tumor	52.1 ± 2.3^###^	43.3 ± 3.1^###^	1.88 ± 0.23^###^	47.1 ± 1.3^###^
T+ 6.25	43.8 ± 1.8	38.6 ± 2.6	1.21 ± 0.21	44.5 ± 1.8
T+ 12.5	41.3 ± 3.1	38.2 ± 2.4	0.85 ± 0.18	32.3 ± 0.5
T+ 25	38.9 ± 2.9*	33.2 ± 2.8*	0.71 ± 0.16*	32.2 ± 1.1**
T+ 50	36.6 ± 1.6*	31.8 ± 2.9**	0.68 ± 0.11*	27.1 ± 1.4**

Hepatic and renal parameters were assessed in both normal (Normal) and untreated tumor-bearing mice (Tumor), and in mice with tumors that were treated with varying doses of artemisinin (T+6.25, 12.5, 25 and 50 mg/kg bodyweight). There was a significant increase in serum biochemical parameters in the Tumor when compared to the Normal. This aberrant increase was restored in ART treated tumor-bearing mice, significantly in T+25 and T+50, when compared to the Tumor. ^###^p<0.001 compared to the Normal and ^*^p<0.05, ^**^p<0.01 compared to the Tumor as determined by Student’s t-test, (n=5) in each case. Each analysis was performed three times.

Analyses of hematological parameters revealed that there were no significant alterations in parameters like hemoglobin (**Figure 1E**), RBC count (**Figure 1F**) and WBC count (**Figure 1G**) in normal mice treated without or with ART (**Table 3**). However, tumor-bearing mice showed anemia-like conditions as indicated by reduction in hemoglobin concentration, which were nevertheless reverted to normal values on ART treatment (**Figure 1E**). The RBC counts reduced significantly (p<0.001), concomitant with an increase in the number of WBCs in tumor-bearing mice compared to normal mice (**Table 4**). All these aberrations were reverted to normal values upon ART treatment (**Figures 1F, G**). Overall, a Kaplan-Meier plot indicated improved mean survival of tumor-bearing mice treated with ART up to almost 90 days compared to tumor-bearing mice which were not exposed to ART and succumbed within a period of 50 days (**Figure 1H**; **Table 5**).

**Table 3:** ART treatment did not alter hematological parameters in normal mice.

ART mg/kg body wt	Hb (g/dl)	Total RBC (10^6^cells/mm^3^)	Total WBC (10^3^ cells/mm^3^)	Differential Count %				
				N	L	M	E	B
Normal	14.8± 0.13	8.8± 0.41	9.3± 2.4	31± 4.8	68± 3.6	1	0	0
6.25	13.1± 0.45	8.1± 0.62	8.7.8± 2.1	33± 3.1	67± 4.1	0	0	0
12.5	14.1± 0.47	7.8± 0.38	8.3± 1.8	28± 3.5	72± 1.7	0	0	0
25	14.7± 0.39	8.2± 0.34	8.75± 2.6	26± 2.3	74± 3.4	0	0	0
50	13.8± 0.41	8.1± 0.41	8.3± 1.44	29 ± 3.2	70± 2.2	1	0	0

Hemoglobin content (Hb), Red Blood Corpuscles (RBC) and White Blood Corpuscles (WBC) count upon ART treatment at different doses (6.25, 12.5, 25 and 50 mg/kg bodyweight) in normal mice, did not show any significant aberrations (n=5), compared to untreated normal mice (Normal). Differential count also did not reveal any variations. N - Neutrophils; L - Lymphocytes; M - Monocytes; E - Eosinophils, and B - Basophils.

**Table 4:** ART treatment reverts aberrations in hematological parameters of tumor-bearing mice to normal values.

ART mg/kg body wt	Hb (g/dl)	Total RBC (10^6^ cells/mm^3^)	Total WBC (10^3^ cells/mm^3^)	Differential Count %				
				N	L	M	E	B
Normal	14.8± 0.13	8.8± 0.41	9.3± 2.4	31± 4.8	68± 3.6	1	0	0
Tumor	7.8± 0.63^###^	4.8± 0.12^###^	28.3± 1.4^###^	71± 2.3^###^	27± 1.7^###^	1	1	0
T+ 6.25	10.1± 0.42	5.1± 0.1	16.8± 0.8**	63± 1.4	36± 2.5	1	0	0
T+ 12.5	13.7± 0.33^**^	6.3± 0.29^*^	10.2± 1.16^***^	48± 2.6^**^	51± 2.6^**^	1	0	0
T+ 25	14.2± 0.21^**^	6.7± 0.14^*^	9.3± 1.8^***^	46± 1.3^**^	53± 2.1^**^	1	0	0
T+ 50	14.6± 0.61^**^	7.6± 0.67^*^	9.1± 1.23^***^	41± 1.5^**^	57± 1.2^**^	2	0	0

Untreated tumor bearing mice (Tumor) showed significant reduction (p<0.001) in hemoglobin content (Hb) and Red Blood Corpuscles (RBC) and significant increase in White Blood Corpuscles (WBC) count (p<0.001), compared to normal mice (Normal). Differential count revealed significant increase in neutrophil count (p<0.001) and significant decrease in lymphocyte count (p<0.001) in Tumor compared to the Normal. Upon ART treatment at different doses (T+6.25, T+12.5, T+25 and T+50 mg/kg bodyweight) in tumor-bearing mice, these alterations significantly reverted to the normal values in a dose-dependent manner^. ###^p<0.001 compared to the Normal and ^*^p<0.05, ^**^p<0.01, ^***^p<0.001 compared to the Tumor; n=5 in each case. N - Neutrophils; L- Lymphocyte; M - Monocytes; E - Eosinophils, and B - Basophils.

**Table 5:** ART treatment increased Mean Survival Time (MST) in tumor-bearing mice.

ART mg/kg body wt	MST (days)
Normal	>90
Tumor	32 ± 13.2^###^
T+ 6.25	48.2 ± 17.3
T+ 12.5	61.3 ± 8.9
T+ 25	75.4 ± 7.1^***^
T+ 50	88.3 ± 8.9^***^

The MST of untreated tumor-bearing mice (Tumor) significantly reduced (p<0.001) compared to the normal mice (Normal). Upon ART treatment at different doses (T+6.25, T+12.5, T+25 and T+50 mg/kg body weight) in tumor-bearing mice, the MST increased in a dose-dependent manner, more significantly (p<0.001) in T+25 and T+50 groups, compared to the Tumor. ^###^p<0.001, compared to the Normal; ^***^p<0.001 compared to the Tumor; n=5 in each case.

### 3.2 Artemisinin disrupts tissue architecture and induces cell death in mice tumors

**Figure 2:**
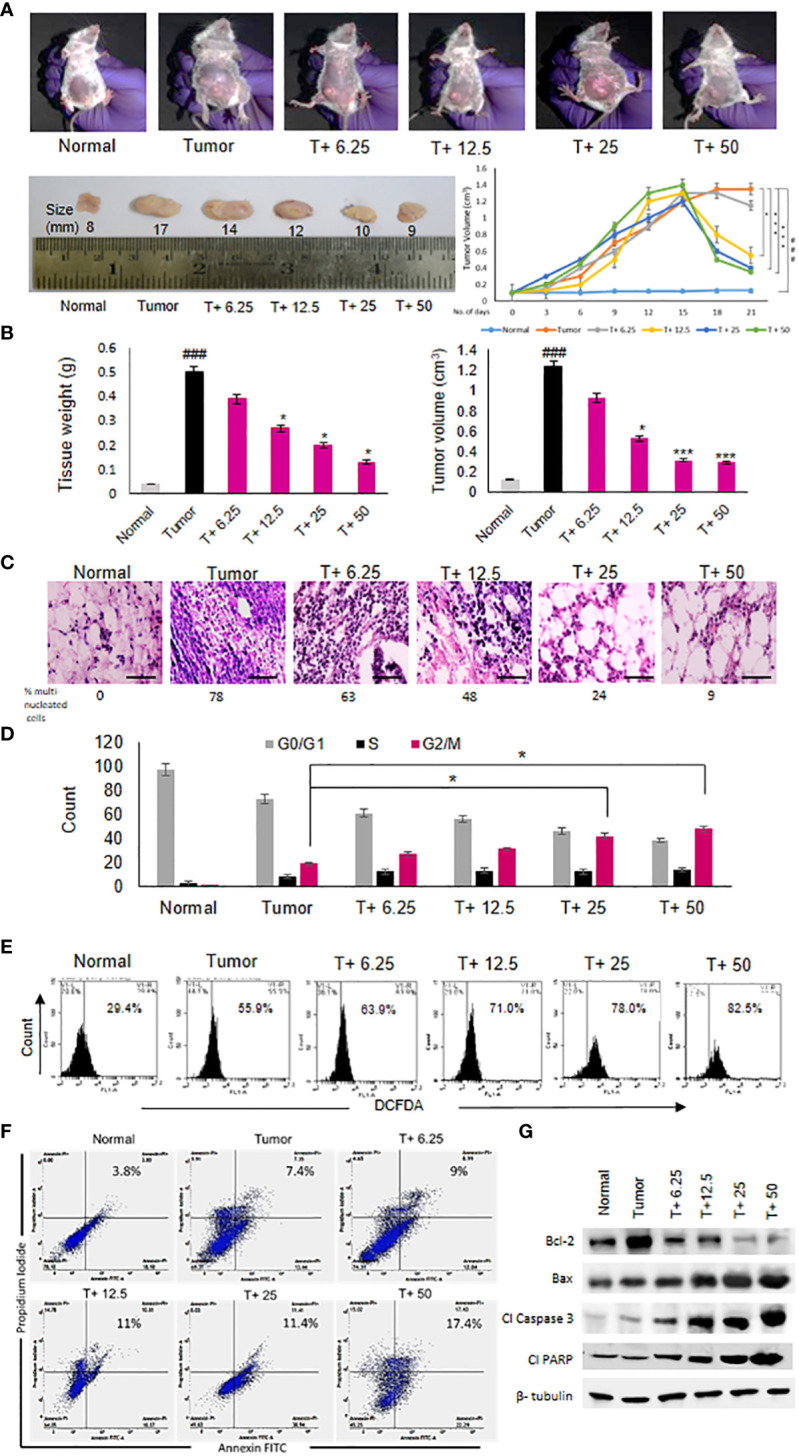
Artemisinin shows anti-cancer effects and induces tumor cell death. **(A)**, Reduction in the size of mammary tumors was observed from 17 mm to 9 mm after ART treatment. Tumor-bearing mice (T+ 6.25, T+12.5, T+25 and T+50) showed reduction in tumor volume from Day 15 onwards, after commencement of ART treatment. **(B)**, Weight and volume of the tumors increased significantly (p<0.001) compared to normal mammary fat pad tissues of untreated mice. There was a significant reduction in tumor weight (p<0.05) and volume (p<0.001) of ART-treated mice. **(C)**, Hematoxylin and eosin staining indicated disruption of tissue architecture and increased number of aggregated multi-nucleated cells in the tumors at 40X magnification (scale bar, 100 µm). ART treatment reduced the number of aggregated multi-nucleated cells in a dose-dependent manner. **(D)**, Cell cycle analyses by flow cytometry revealed significant increase (p<0.05) in the G2/M population compared to untreated tumor-bearing mice, indicating G2/M cell cycle arrest, specifically when mice were exposed to 25 and 50 mg/kg ART. **(E)**, ROS generation in tumors, indicated a dose-dependent increase in ROS from 55.9% in tumor cells to 82.5% in 50 mg/kg ART treated mice. **(F, G)**, Apoptotic cells were assessed using flow cytometry **(F)** and western blot analyses **(G)**. Significant increase in the percentage of apoptotic cells was observed in response to ART treatment compared to the untreated tumor. Immunoblotting for apoptotic markers revealed a dose-dependent decrease for Bcl-2 and increase for pro-apoptotic markers, such as Bax, cleaved caspase 3 and cleaved PARP compared to the naïve tumor. The relative protein expressions were normalized using β-tubulin. ^###^p<0.001 compared to normal mice, ^*^p<0.05 and ^***^p<0.001 compared to the tumor; n=5. Data are presented as mean ± SD and are representative of three independent experiments.

ART treatment reduced tumor diameter in response to varying doses compared to the untreated tumor (**Figure 2A**). Significant decrease in tumor weight and volume was observed in tumor tissues (**Figure 2B**) of tumor-bearing mice treated with the doses of 12.5 mg/kg, 25 mg/kg and 50 mg/kg body weight of ART compared to the untreated tumor-bearing mice. Histopathological analyses revealed regular tissue architecture of mammary fat pad of normal mice. Tumor tissue showed disruption of regular tissue architecture and accumulation of numerous multi-nucleated tumor cells (**Figure 2C**). Upon ART treatment of tumor-bearing mice it was observed that the number of multi-nucleated cells counted per 100 fields reduced and the tissue arrangement appeared less distorted in response to increasing doses of treatment (**Figure 2C**). PI staining of cells isolated by collagenase-hyaluronidase digestion from normal tissue and tumor tissues with or without ART treatment was performed to estimate the DNA content of these cells. It was observed that a significant increase in the percentage of G_2_/M cell count (p<0.05) in 25mg/kg and 50 mg/kg bodyweight ART-treated tumor bearing mice compared to the G_2_/M cell count of untreated tumor-bearing mice (**Figure 2D**). DCFDA staining of normal and tumor cells with or without ART treatment was performed to estimate the levels of reactive oxygen species (ROS) in these cells. A dose-dependent increase in percentage of cells generating ROS was observed upon ART treatment in tumor-bearing mice compared to the untreated tumor (**Figure 2E**). Annexin-FITC and PI staining was performed to estimate apoptotic cell population in normal mammary fat pad and tumor cells. The result reveals an increase in percentage of apoptotic cells in tumor cells of ART-treated tumor-bearing mice in a dose-dependent manner (**Figure 2F**). To further validate this data, western blot analysis was performed, and a decrease in expression of anti-apoptotic protein (Bcl-2) and an increase in expression of pro-apoptotic proteins (Bax, cleaved caspase 3 and cleaved PARP) was evident in tumor tissues of ART-treated tumor-bearing mice compared to the untreated tumor-bearing mice (**Figure 2G**).

### 3.3 Artemisinin reduces CLEC12A expression and represses autophagy in tumor cells

**Figure 3:**
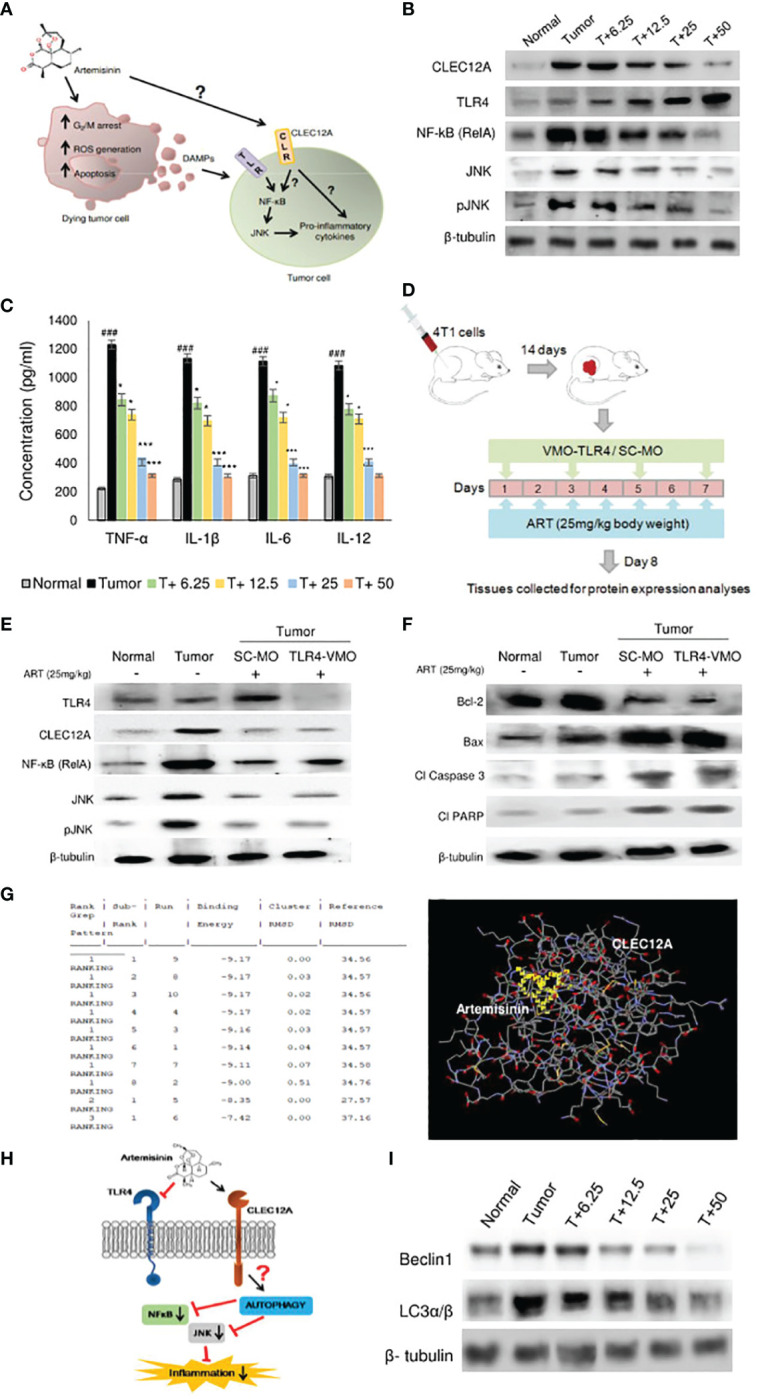
Artemisinin directs CLEC12A through an alternate pathway to reduce inflammation in mice tumors. **(A)**, Diagrammatic representation of the possible role of ART on tumor progression and apoptosis. ART may directly affect certain cells of tumors, leading to G2/M cell cycle arrest, ROS generation and apoptosis. Dead cells secrete DAMPs that can be sensed by PRRs of surrounding tumor cells, where they promote inflammation and augment tumorigenesis. The role of ART on these tumor cells needs to be established. **(B)**, Expression of CLEC12A decreased and TLR4 increased in response to ART treatment compared to untreated tumor tissue. Unconventionally, expressions of NF-κB (RelA) and JNK/pJNK reduced in response to increasing doses of ART. The relative protein expressions were normalized using β-tubulin. **(C)**, Estimation of serum pro-inflammatory cytokines revealed significant increase in the untreated tumor, which significantly decreased in ART-treated tumor- bearing mice. **(D)**, Scheme depicting TLR4 knockdown attained by vivo-morpholino oligomers (VMO) specific for TLR4. **(E)**, Silencing TLR4 with TLR4-VMO in tumor-bearing mice completely diminished its expression after ART treatment. There was no change in the expressions of CLEC12A, NF-κB (RelA) and JNK/pJNK between SC-MO and TLR4-VMO treatment in mice when exposed to 25 mg/kg ART. **(F)**, Expressions of apoptotic markers in tumors did not reveal any change between SC-MO and TLR4-VMO groups upon 25 mg/kg ART treatment. **(G)**, *in silico* docking analysis implied a possible interaction between CLEC12A and ART with a negative ΔG value (ΔG= -9.17). **(H)**, A schematic representation of a possible non-canonical pathway that CLEC12A may adopt in response to ART, whereby downregulation of CLEC12A by ART might alter autophagy to eventually restrict the inflammatory cascade mediated by NF-κB (RelA) and JNK/pJNK. **(I)**, Immunoblot demonstrating the expressions of autophagic markers (Beclin-1 and LC3α/β) in response to different concentrations of ART compared to untreated tumor. The relative protein expressions were normalized with β-tubulin. ^###^p<0.001; compared to normal mice, ^*^p<0.05; ^***^p<0.001; compared to tumor-bearing mice not treated with ART. Data are presented as mean ± SD and are representative of three independent experiments; n=5 in each case.

To further ascertain the targets and intracellular role of ART (**Figure 3A**), its effects on CLEC12A and downstream components were investigated. Immunoblots were first performed to investigate the expression of CLEC12A in normal mammary fat pad, tumor tissues from untreated tumor-bearing mice and ART-treated tumor-bearing mice. Mice with tumors revealed enhanced expression of CLEC12A by almost 4-fold (p<0.01) compared to normal mice. However, the expression of CLEC12A reduced upon ART treatment in tumor tissues in a dose-dependent manner (**Figure 3B**). Since TLR4 is known to be suppressed by CLEC12A, the expression of TLR4 was reduced in tumor tissues and gradually increased with ART treatment (**Figure 3B**). However, contradictory to our expectations, we observed a decrease in expression of NF-kB, JNK and pJNK in ART-treated mice compared to the untreated mice bearing tumors (**Figure 3B**). Concomitantly, a sandwich ELIZA for pro-inflammatory cytokines (TNF-α, IL-1β, IL-6 and IL-12) revealed a dose-dependent reduction in levels of pro-inflammatory cytokines (p<0.05 and p<0.001) of ART-treated tumor-bearing mice compared to the untreated tumor-bearing mice (**Figure 3C**). To justify the possible non-involvement of TLR4 in this pathway, we silenced TLR4 in mice using vivo morpholino oligomers against TLR4 mRNA (TLR4-VMO). As a control, tumor-bearing mice were treated with a scramble morpholino oligomer (SC-MO). Mice were simultaneously treated with 25 mg/kg ART, as described in **Figure 3D**. The results revealed that TLR4 expression significantly diminished (p<0.001) in TLR4-VMO compared to the SC-MO. Inhibition of TLR4 along with ART co-treatment, however, did not alter the expression of CLEC12A, NF-κB (RelA) or JNK/pJNK (**Figure 3E**). In addition, co-treatment with ART did not alter expression of apoptotic markers *in vivo* morpholino-treated mice compared to scrambled morpholino-treated tumor-bearing mice (**Figure 3F**). In order to establish a direct interaction between CLEC12A and ART, an in silico docking study was performed, using the AutoDock 4.2.6 software. Computational docking was performed ten times and any possible receptor-ligand interaction was analyzed. The results indicated that ART interacts directly with CLEC12A. Binding occurred with a spontaneous negative binding energy, the lowest energy value being -9.17 (**Figure 3G**). Further, ART interacts with CLEC12A around residues Ser184, Ser186, Tyr187, Asp188 and Arg232, which lies in the ligand-binding surface of CLEC12A, specifically in its CDD domain, as determined from InterPro database (**Figure 3G**). Since ART prevented CLEC12A-TLR4 interaction, an alternative pathway involving autophagy was proposed, which might down-regulate inflammation (**Figure 3H**). Western blot analyses indicated that ART reduced autophagy in a dose-dependent manner, as confirmed by decreased expression of autophagic markers, beclin1 and LC3α/β (**Figure 3I**; **Supplementary Figure 2**).

### 3.4 Overexpression of CLEC12A followed by artemisinin treatment enhances autophagy and inflammatory response in TNBC cells

**Figure 4:**
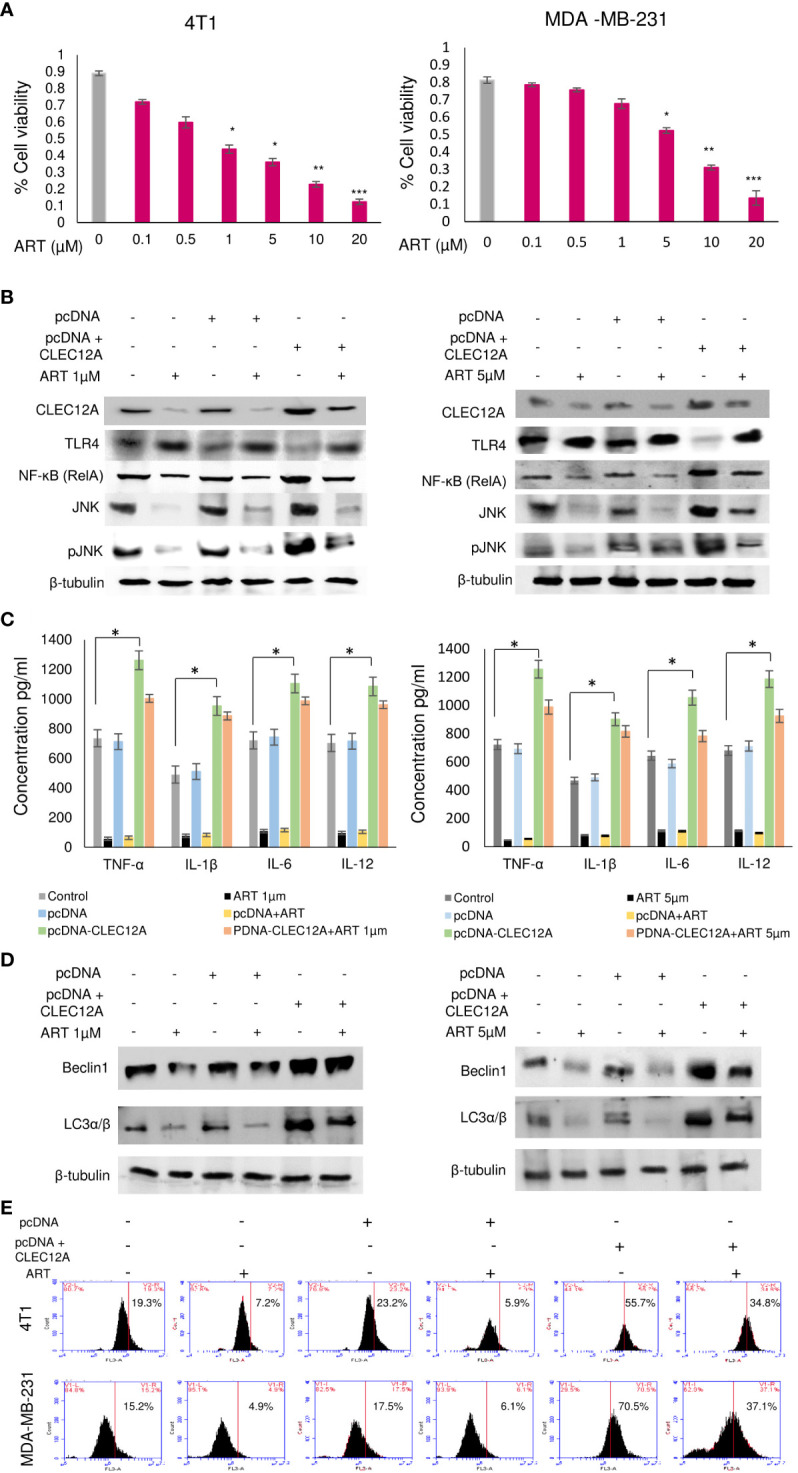
Overexpression of CLEC12A countermands the anti-cancer effects of artemisinin. **(A)**, Cell viability in response to ART treatment was determined in 4T1 and MDA-MB-231 cells. IC50 values, determined to be 1 µM in 4T1 cells and 5 µM in MDA-MB-231 cells, were selected for subsequent studies. **(B)**, CLEC12A was overexpressed in 4T1 and MDA-MB-231 cells, followed by treatment without or with ART. Immunoblotting confirmed enhanced expression of CLEC12A by 2-fold in CLEC12A-overexpressed cells compared to the non-transfected control and pcDNA vector transfected cells. ART treatment did not reduce CLEC12A expression in CLEC12A-overexpressed cells as it diminished the level in non-transfected control and pcDNA vector transfected cells. The expression of TLR4 reduced and that of NF-κB (RelA) and JNK/pJNK increased in CLEC12A overexpressed cells compared to the non-transfected control and pcDNA vector control. ART treatment of CLEC12A-overexpressed did not restore levels of TLR4, NF-κB and JNK/pJNK to those observed in non-transfected control and pcDNA vector controls. **(C)**, Levels of pro-inflammatory cytokines significantly elevated (p<0.05) in CLEC12A-overexpressed cells and ART treatment did not revert to the levels of non-transfected and pcDNA vector transfected cells. **(D)**, Expression of autophagic markers (Beclin1 and LC3α/β) increased in CLEC12A-overexpressed cells and remained relatively high after ART treatment in non-transfected and pcDNA vector-transfected cells. **(E)**, Additionally, the percentages of autophagic cells were analyzed by flow cytometry and a similar trend was observed where overexpression of CLEC12A enhanced the percentage of autophagic cells and ART treatment did not reduce the percentage to those observed in non-overexpressed and pcDNA vector-transfected cells. ^*^p<0.05; ^**^p<0.01; ^***^p<0.001. The relative protein expressions were normalized with β- tubulin. Data are presented as mean ± SD and are representative of three independent experiments.

In order to further validate our findings, we overexpressed CLEC12A in murine mammary cancer cells (4T1) and human breast cancer cells (MDA-MB-231) and checked the effects in response to ART treatment. Primarily, we estimated the IC_50_ values of ART in both cell lines in response to varying doses of ART. It was found that ART IC_50_ value in 4T1 cells was 1 μM and in MDA-MB-231 cells was 5 μM (**Figure 4A**). These doses were used for all subsequent experiments. Immunoblots confirmed that pcDNA-CLEC12A transfected cells have significantly higher expression (p<0.001) of CLEC12A compared to non-transfected cells and empty vector transfected cells. Moreover, in pcDNA-CLEC12A transfected cells, the expressions of NF-kB, JNK and pJNK was significantly higher (p<0.001) compared to non-transfected and empty vector transfected cells. ART treatment significantly reduced the expressions of the above components (**Figure 4B**), in both the cell lines. It was also observed that pcDNA-CLEC12A transfected cells have significantly enhanced levels (p<0.001) of pro-inflammatory cytokines compared to non-transfected cells and empty vector transfected cells (**Figure 4C**), and ART treatment did not reduce the levels of the cytokines as it did for non-transfected and empty vector transfected cells. In addition, the pcDNA-CLEC12A transfected cells showed significantly elevated expression (p<0.001) of autophagic markers, beclin1 and LC3αβ, compared to non-transfected cells and empty vector transfected cells whereas ART treatment significantly reduced their expressions (**Figure 4D**). The immunoblot data was further confirmed by acridine orange staining of acidic vesicular organelles (AVOs) which indicated that pcDNA-CLEC12A transfected cells have a higher percentage of AVOs in cells compared to non-transfected cells and empty vector transfected cells. ART treatment reduced the percentage of AVOs in pcDNA-CLEC12A transfected cells in both cell lines (**Figure 4E**).

### 3.5 CLEC12A over expression suppresses artemisinin-induced apoptosis

**Figure 5:**
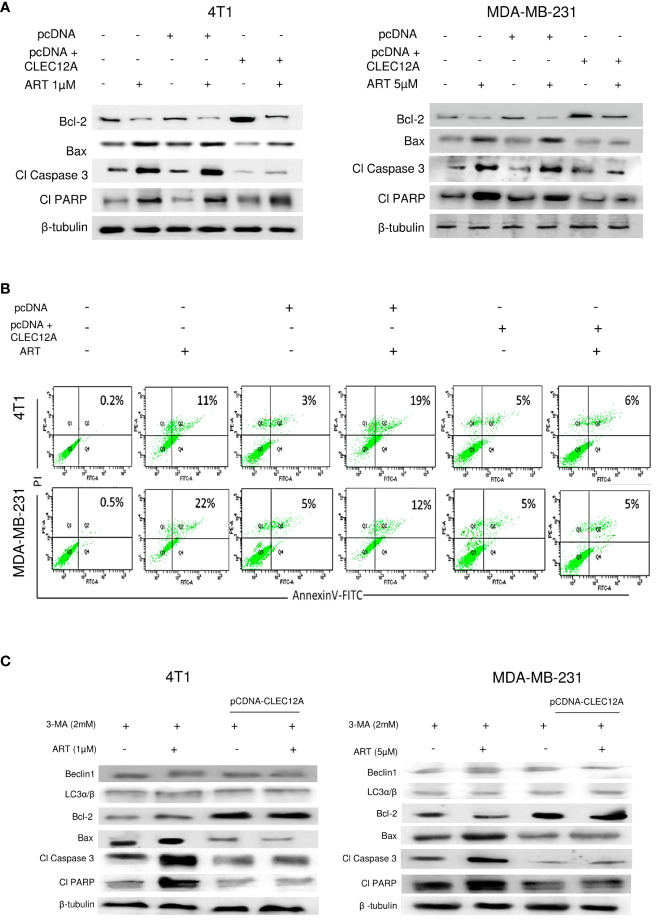
CLEC12A overexpression restricts the ability of artemisinin to induce apoptosis. **(A)**, Immunoblotting revealed that CLEC12A overexpressed cells showed increased expression of Bcl-2 and reduced expressions of Bax, cleaved caspase 3 and cleaved PARP, compared to the non-transfected control and pcDNA vector control cells. Upon ART treatment of CLEC12A overexpressed cells, the expressions of the apoptotic markers did not conform to the levels observed in ART-treated non- transfected and pcDNA vector-transfected cells. **(B)**, The percentage of apoptotic cells was estimated by flow cytometry. In the presence of ART, only 5% cells underwent apoptosis in CLEC12A-overexpressed 4T1 cells, compared to 22% in non-transfected control and 19% in vector control cells. Similarly, in MDA-MB-231 cells, only 2% cells underwent apoptosis upon ART treatment in CLEC12A-overexpressed cells, compared to 11% in non-transfected control and 12% in vector control cells. **(C)**, ART-induced apoptosis was determined after treating cells with an autophagy inhibitor, 3-methyladenine (3-MA), in non-overexpressed and CLEC12A-overexpressed 4T1 and MDA-MB-231 cells. The expressions of Beclin1 and LC3α/β remained low under all conditions. Compared to non-overexpressed cells, those with overexpressed CLEC12A showed low expression of pro-apoptotic markers. ART treatment did not induce apoptosis in the autophagy-inhibited CLEC12A-overexpressed cells compared to non-overexpressed cells, as evident by reduced expressions of pro-apoptotic markers. The relative protein expressions were normalized with β- tubulin. Data are presented as representative of three independent experiments.

Immunoblotting revealed increased (p<0.001) expression of the anti-apoptotic protein (Bcl-2) in pcDNA-CLEC12A transfected cells compared to non-transfected cells and empty vector transfected cells, concomitant with reduced expressions of pro-apoptotic markers (Bax, cleaved caspase 3 and cleaved PARP). ART treatment, however, failed to elevate the expressions of the above proteins in pcDNA-CLEC12A transfected cells, compared to the non-transfected and empty vector transfected cells. Similar results were observed in both the murine and human cell lines (**Figure 5A**). To further confirm the immunoblot data, Annexin-FITC and PI staining was performed to estimate apoptotic cell population by flow cytometry. It was observed that the percentage of apoptotic cells increased (0.2% to 11% in 4T1 cells and 0.5% to 22% in MDA-MB-231 cells; p<0.001) in response to ART treatment in both cell lines. However, ART treatment did not substantially elevate the percentage of apoptotic cells in pcDNA CLEC12A transfected cells (**Figure 5B**). In order to validate whether ART treatment downregulates CLEC12A and autophagy to induce apoptosis in tumor cells, cells were treated with the autophagy inhibitor 3-methyladenine (3-MA) followed by treatment with ART in normal and CLEC12A-overexpressed cells. Western blot analysis revealed that upon 3-MA treatment of pcDNA-CLEC12A overexpressed cells, the levels of anti-apoptotic protein (Bcl-2) was significantly higher (p<0.001) in both cell lines compared to non-transfected cells. Upon ART treatment, the expression of Bcl-2 remained significantly higher (p<0.001) in pcDNA-CLEC12A cells compared to ART treatment of untransfected cells. Concomitantly, on ART treatment, the expression of pro-apoptotic markers (Bax, cleaved caspase 3 and cleaved PARP) remained significantly low (p<0.001) as compared to ART treatment of untransfected cells (**Figure 5C**).

### 3.6 Artemisinin targets cancer stem cells in mammary tumor cells

**Figure 6:**
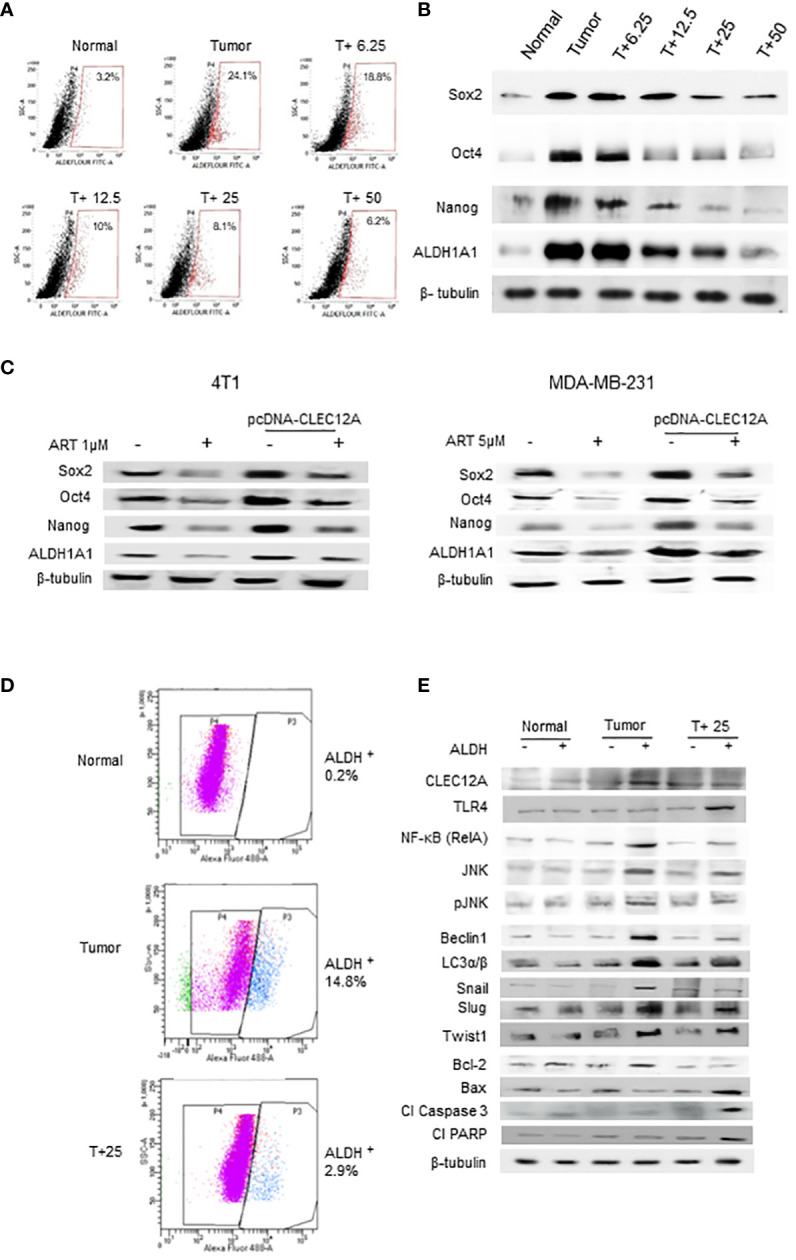
Artemisinin targets the cancer stem cell population in mammary tumors and breast cancer cell lines. **(A)**, Aldefluor assay revealed a 7.5-fold increase in the CSCs in mice tumors compared to normal tissues. ART treatment reduced the percentage of ALDH+ cells in a dose-dependent manner, by almost 4-fold in the 50 mg/kg treated mice (p<0.01). **(B)**, Expressions of CSC markers (Sox2, Oct4, Nanog and ALDH1A1), determined by western blot analyses, revealed a dose-dependent reduction in response to ART treatment compared to the untreated tumor. **(C)**, Expressions of CSC markers were elevated in CLEC12A-overexpressed 4T1 and MDA-MB-231 cells compared to the non-overexpressed cells. ART treatment reduced expression of CSC markers in CLEC12A-overexpressed cells, but comparatively less as that observed in ART-treated non-overexpressed cells. **(D)**, Cancer cells (ALDH^-^) and CSCs (ALDH^+^) were sorted from normal mammary tissues, untreated tumors and tumors from mice treated with 25 mg/kg body weight of ART. A 5-fold (p<0.01) reduction was observed in CSCs after ART treatment. **(E)**, Relative protein expressions of CLEC12A, TLR4, NF-κB, JNK/pJNK, and proteins related to autophagy, EMT and apoptosis were compared in the cancer cells versus CSCs in the sorted cell populations. Expression of CLEC12A, NF-κB (RelA), JNK/pJNK, Beclin1 and LC3α/β, Snail, Slug and Twist1 were conspicuously high in the ALDH^+^ population, whereas that of TLR4 and apoptotic markers were low compared to the ALDH^-^ population. Expressions of all the above were reversed on ART treatment, specifically in the ALDH^+^ population. Relative protein expressions were normalized with β-tubulin.

Since a tumor mass comprises a heterogenous population of cells, including the quiescent and drug-resistant cancer stem cells (CSCs), we analyzed the effects of ART in the CSCs. Aldefluor™ staining and analysis revealed significant reduction (up to 8.1% in 25 mg/kg and 6.2% in 50 mg/kg ART/body weight-treated mice; p<0.001) in the percentage of ALDH^+^ cells compared to CSCs in the untreated tumor (24.1%; **Figure 6A**). In confirmation, immunoblotting revealed a dose dependent reduction in expression of CSC makers (Sox2, Oct4, Nanog and ALDH1A1) in the ART-treated tumors compared to the untreated tumor (**Figure 6B**). Moreover, in both 4T1 and MDA-MB-231 cells, we found that the expression of these CSC markers were significantly higher (p<0.001) in pcDNA-CLEC12A transfected cells compared to non-transfected cells. However, upon ART treatment, reduction in expression levels was observed (**Figure 6C**). Next, cells were sorted using ALDH from mammary fat pad (Normal), tumor tissues from untreated tumor-bearing mice (Tumor) and 25 mg/kg ART-treated tumor bearing mice (T+25; **Figure 6D**). The results revealed that the expression of CLEC12A was higher in ALDH^+^ population of Tumor, TLR4 was higher in ALDH^+^ population of T+25. NF-kB, JNK and pJNK showed relatively higher expressions in ALDH^+^ of Tumor, but these expressions reduced in ALDH^+^ of T+25. Similar trend was observed in the expression of autophagic markers, which reduced on ART treatment. This indicates a similar pathway is observed in CSCs. Moreover, EMT markers (Snail, Slug and Twist1) which are indicative of the metastatic potential in CSCs were high in ALDH^+^ population of Tumor, but it decreased in the ALDH^+^ population in T+25. Finally, anti-apoptotic marker, Bcl-2 was higher in ALDH^+^ population of Tumor but decreased in ALDH^+^ of T+25. All pro-apoptotic markers were elevated in ALDH^+^ population of T+25 (**Figure 6E**). These above findings prove that ART downregulates CLEC12A and autophagy to induce apoptosis in both tumor cells, as well as CSCs.

## 4 Discussion

Intra-tumoral heterogeneity is a major caveat in the treatment of breast cancer, leading to poor prognosis and reduced survivability of patients (49). It is a major cause of treatment failure since different cells within a tumor have selective sensitivities to anti-cancer drugs (50). In addition, genetic/epigenetic and environmental factors affect the persistence of the invincible CSC population, which increases the risk of therapy resistance and tumor recurrence (21). Such hierarchy bestows additional responsibility on anti-cancer drugs to target the diverse populations of cells within a tumor with extra proficiency.

Over the years, ART gained popularity for its anti-cancer, as well as, immunomodulatory and anti- inflammatory effects (18). In particular, treatment of normal mice with ART revealed no adverse effects on normal cells. Therefore, developing ART as a possible therapeutic drug would be beneficial since it should pose minimal side effects in patients with tumors. In tumor-bearing mice, where significant adversities were observed in hematological parameters and primary metastatic organs like liver, kidney and lungs, ART successfully reversed the disrupted physiological parameters towards normalcy. In addition, ART showed potential anti-cancer effects on the tumor cells, like reduction in the number of multi-nucleated cells, G_2_/M cell cycle arrest, enhanced ROS generation and increased apoptosis.

Radiation and chemotherapy induce apoptosis of rapidly proliferating tumor cells; as a consequence, damage-associated molecular patterns (DAMPs) are released from dying tumor cells. This triggers immune cells or persevering CSCs to activate pattern-recognition receptors (PRRs) and orchestrate an inflammatory response (51), by promoting the release of pro-inflammatory cytokines (52). Thus, DAMPs from different cells affect inflammation and tumor progression (53, 54), primarily by binding to TLRs. It is thereby essential for a putative anti-cancer drug to prevent activation of TLRs and inhibit an inflammatory response. Since ART exhibited definitive anti-cancer effects on the mammary tumor cells, we attempted to delineate its effects specifically on the CSCs. In addition, any involvement of CLEC12A in response to ART treatment that can modulate the resilient CSCs and lead them towards apoptosis was also investigated. In human monocytes and granulocytes, CLEC12A, an inhibitory PRR, restrains the activation cascade initiated by the TLRs (55). Conventionally, activation of TLRs, which are also found on tumor cells of non-hematopoietic origin, can lead to heightened inflammation and tumor progression (3). Therefore, the hypothesis was that inhibition of TLR4 by ART-activated CLEC12A would prevent inflammation and restrain tumor progression, since we observed anti-cancer effects of ART in our primary mice studies.

The paradox was encountered when further in-depth analyses revealed that contrary to our hypothesis, ART significantly downregulated CLEC12A expression, and increased the expression of TLR4 in the presence of ART in tumor tissues. This finding prompted us to explore alternative downstream signaling components which led to death of tumor cells. Surprisingly, although the expression of TLR4 increased, the expressions of TLR4-downstream components NF-κB (RelA) and JNK/pJNK reduced in response to ART treatment. These results were in direct contradiction to the established fact that canonical signaling via increased TLR4 should have classically augmented NF-κB (RelA) expression, leading to increased inflammation in tumor cells (51, 56), and eventually resistance to ART. This led to the assumption that although ART inhibited expression of CLEC12A, it did not affect the downstream events in a tumor cell via TLR4. Therefore, in order to confirm the assumption and essentially rule out involvement of TLR4 in ART-induced tumor cell death, TLR4 was silenced in tumor-bearing mice using vivo-morpholino. The results indicated that ART continued to reduce the expression of CLEC12A in TLR4-silenced mice, since it was upstream of TLR4. However, expressions of NF-κB (RelA) and JNK/pJNK reduced in response to ART in both TLR4-expressed and silenced tumor-bearing mice, indicating that TLR4 was not involved in the anti-cancer effects of ART. Concomitantly, ART induced apoptosis of tumor cells, irrespective of the status of TLR4, leading us to assume that ART adopted a non-canonical pathway which differentiated the role of CLEC12A in solid tumors from that known in myeloid cells (11).

Search for an alternative pathway led to a finding which stated that CLEC12A may play a role in autophagy in defense pathways, and downregulation of CLEC12A may lead to inhibition of autophagy (14). Therefore, reduced expression of CLEC12A by ART may affect autophagy and suppress pro-inflammatory responses in tumors, bypassing the conventional TLR4 pathway. In accordance, our results indicated a dose-dependent reduction in autophagy-related proteins in response to ART. Interestingly, it is well established that inhibition of autophagy can suppress NF-κB-mediated inflammation (57), which further supports a non-canonical pathway adopted by ART through which it downregulates CLEC12A, inhibits autophagy, decreases expressions of NF-κB (RelA) and JNK/pJNK, and reduces inflammation in the tumor cells, eventually culminating in cell death. In order to confirm this pathway, we overexpressed CLEC12A using a pcDNA 3.1(+) expression vector in both murine 4T1 and human MDA-MB-231 triple negative breast cancer cells. Over expression of CLEC12A reduced TLR4 expression, enhanced expressions of NF-κB (RelA) and JNK/pJNK, and increased the concentration of pro-inflammatory cytokines, viz., TNF-α, IL-1β, IL-6 and IL-12, in CLEC12A-overexpressed cells in contrast to cells where CLEC12A expressed at physiological levels. The rationale for measuring the above cytokines was that the NF-κB family of transcription factors, like RelA, control the direct transcription of TNF-α, IL-1 β, IL-6 and IL-12 pro-inflammatory cytokines (58); hence, the downstream effects were determined by analyzing the levels of these pro-inflammatory cytokines. Treatment with ART marginally ameliorated the levels of the above factors in the CLEC12A-overexpressed cells, but the changes were not significant. Moreover, CLEC12A over expression led to increased autophagy and reduced apoptosis following ART treatment, which did not revert to levels observed in the non-overexpressed cells. This strongly indicated that the anti-proliferative effects of ART were compromised upon CLEC12A over expression, and that downregulation of CLEC12A by ART is a prerequisite for apoptosis of the tumor cells. To further ensure downregulation of autophagy on treatment with ART, autophagy was inhibited with 3-MA in normal and CLEC12A overexpressed cells. The results confirmed that inhibition of autophagy with 3-MA led to ART-induced apoptosis in normal cells, as was also the case when cells were treated with ART. However, in CLEC12A-overexpressed cells, inhibition of autophagy with 3-MA could not induce apoptotic cell death, even in the presence of ART. This further reinstated that CLEC12A downregulation is an absolute necessity for ART-induced apoptosis in mice tumors and breast cancer cells.

Tumor heterogeneity restrains the action of cytotoxic anti-cancer drugs, which may either function temporally on tumor cells at different phases of proliferation (59, 60) or may not permeate the CSCs because of their dormant and chemo-resistant properties (22). Therefore, most conventional drugs have incomplete effectiveness. However, the effect of ART on the relatively quiescent CSCs revealed a dose-dependent decrease in the percentage of ALDH^+^ cells and expression of stem cell markers, assuring that ART is effective in targeting the CSC population, in addition to the cancer cells. Furthermore, on sorting the ALDH^-^ and ALDH^+^ cells from normal, untreated and ART-treated tumors, it was observed that expression of CLEC12A was higher in ALDH^+^ cells of the untreated tumor, and reduced in the CSC population after ART treatment. Concomitantly, NF-κB, JNK/pJNK, autophagy and EMT markers reduced whereas apoptotic markers increased in the CSCs after ART treatment. This study thereby established that in non-hematopoietic cells, CLEC12A bypassed the canonical TLR-mediated signaling and adopted an alternative pathway of restraining inflammation and promoting cell death in the presence of ART, in both mice mammary tumors and breast CSCs. Since ART is already an FDA-approved anti-malarial drug with minimal side effects, it can be repurposed as an effective chemotherapeutic drug for the treatment of breast cancer patients in future.

## Data availability statement

The raw data supporting the conclusions of this article will be made available by the authors, without undue reservation.

## Ethics statement

All procedures involving animals were in accordance with the Principles of Laboratory Animal Care (NIH Publication No 85-23, revised in 1985) and approval of the Institutional Animal Ethical Committee, Government of India (Registration Number 885/ac/05/CPCSEA). Specific Indian Laws of Animal Protection (ILAP) were followed throughout the experimental schedule. The study was conducted in accordance with the local legislation and institutional requirements.

## Author contributions

RC has contributed to investigation, data curation, formal analysis, manuscript-writing, and editing. AS contributed to data acquisition. KC acquired funding. UC conceptualized the study, contributed to funding acquisition, supervision, investigation, formal analysis, interpretation of results, review of work, and editing. All authors contributed to the article and approved the submitted version.
