# Corrigendum: CLEC12A sensitizes differentially responsive breast cancer cells to the anti-cancer effects of artemisinin by repressing autophagy and inflammation

**DOI:** 10.3389/fonc.2024.1393626

**Published:** 2024-03-14

**Authors:** Ranodeep Chatterjee, Aditya Shukla, Kausiki Chakrabarti, Urmi Chatterji

**Affiliations:** ^1^ Cancer Research Laboratory, Department of Zoology, University of Calcutta, Kolkata, India; ^2^ Cell Biology Laboratory, Department of Microbiology, University of Calcutta, Kolkata, India; ^3^ Department of Zoology, Charuchandra College, Kolkata, India; ^4^ Centre for Research in Nanoscience and Nanotechnology, University of Calcutta, Kolkata, India

**Keywords:** Clec12A, artemisinin, TLR4, autophagy, apoptosis, cancer stem cells

In the published article, there was an error in **Figure 6** as published. In **Figure 6E**, multiple β-tubulin blots were incorporated erroneously in between the immunoblot panel. The relative protein expressions in this panel were estimated to their respective β-tubulin expressions at the end of the panel only. The corrected **Figure 6** and its caption appear below.

**Figure 6:**
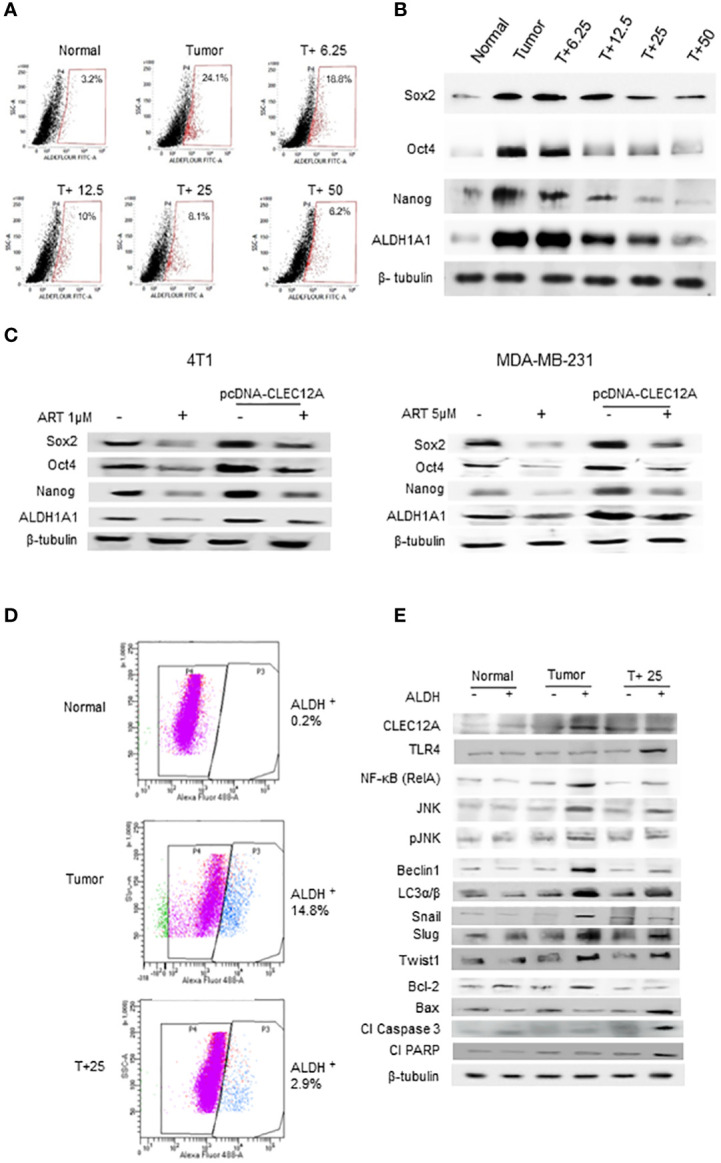
Artemisinin targets the cancer stem cell population in mammary tumors and breast cancer cell lines. **(A)**, Aldefluor assay revealed a 7.5-fold increase in the CSCs in mice tumors compared to normal tissues. ART treatment reduced the percentage of ALDH+ cells in a dose-dependent manner, by almost 4-fold in the 50 mg/kg treated mice (p<0.01). **(B)**, Expressions of CSC markers (Sox2, Oct4, Nanog and ALDH1A1), determined by western blot analyses, revealed a dose-dependent reduction in response to ART treatment compared to the untreated tumor. **(C)**, Expressions of CSC markers were elevated in CLEC12A-overexpressed 4T1 and MDA-MB-231 cells compared to the non-overexpressed cells. ART treatment reduced expression of CSC markers in CLEC12A-overexpressed cells, but comparatively less as that observed in ART-treated non-overexpressed cells. **(D)**, Cancer cells (ALDH-) and CSCs (ALDH+) were sorted from normal mammary tissues, untreated tumors and tumors from mice treated with 25 mg/kg body weight of ART. A 5-fold (p<0.01) reduction was observed in CSCs after ART treatment. **(E)**, Relative protein expressions of CLEC12A, TLR4, NF-kB, JNK/pJNK, and proteins related to autophagy, EMT and apoptosis were compared in the cancer cells versus CSCs in the sorted cell populations. Expression of CLEC12A, NF-kB (RelA), JNK/pJNK, Beclin1 and LC3α/β, Snail, Slug and Twist1 were conspicuously high in the ALDH+ population, whereas that of TLR4 and apoptotic markers were low compared to the ALDH- population. Expressions of all the above were reversed on ART treatment, specifically in the ALDH+ population. Relative protein expressions were normalized with β-tubulin.

The authors apologize for this error and state that this does not change the scientific conclusions of the article in any way. The original article has been updated.

