# Supplementary material for: CLEC12A sensitizes differentially responsive breast cancer cells to the anti-cancer effects of artemisinin by repressing autophagy and inflammation
Source: Front Oncol. 2023 Dec 8;13:1242432. doi: 10.3389/fonc.2023.1242432 (PMC10748408; doi:10.3389/fonc.2023.1242432)
Supplement: Supplementary file 1 [file DataSheet_1.pdf]

Supplementary Material

CLEC12A sensitizes differentially responsive breast cancer cells to the anti-cancer effects of artemisinin by repressing autophagy and inflammation

Ranodeep Chatterjee, Aditya Shukla, Kausiki Chakrabarti, Urmi Chatterji*

Correspondence: Corresponding Author: urmichatterji@gmail.com

Supplementary Figure 1

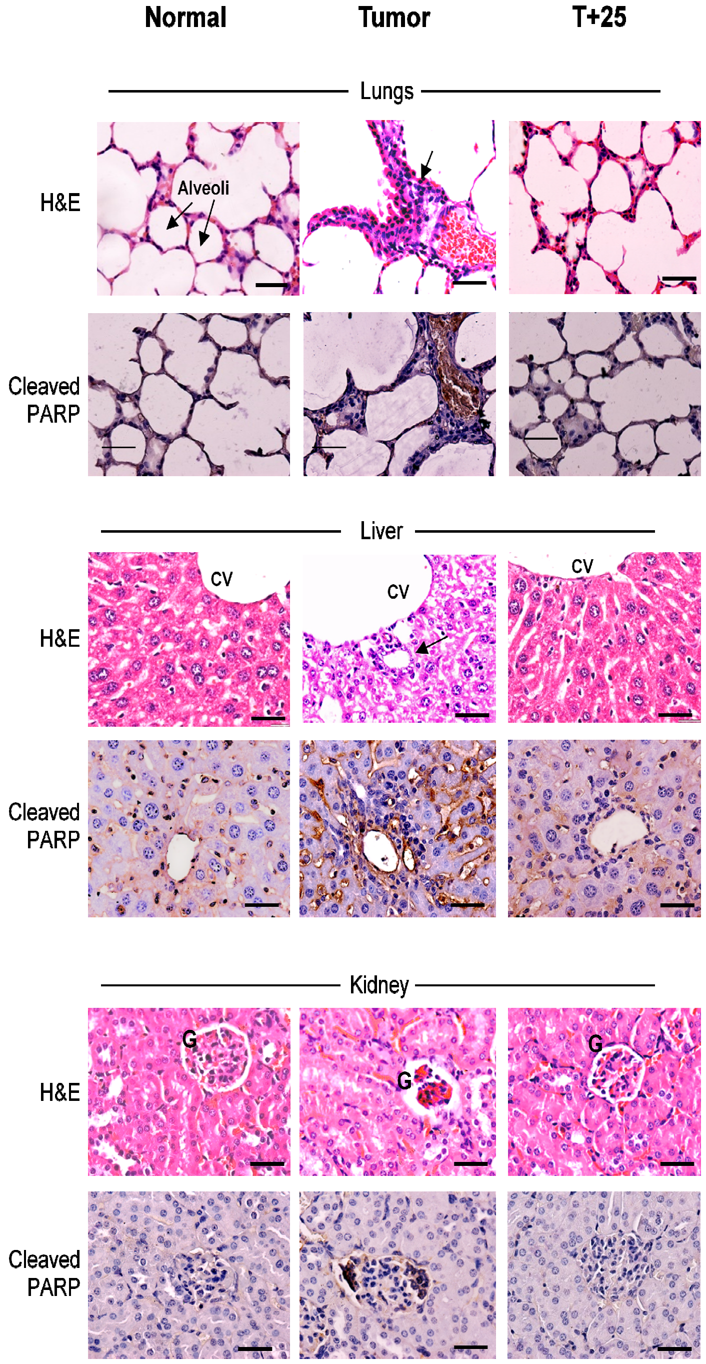

Supplementary Figure 1: Histology and immunohistochemical (IHC) images of tissues from normal, untreated and ART treated tumor-bearing mice. Hematoxylin and eosin staining (H&E) of lung tissue from untreated tumor-bearing mice (Tumor) showed disarrangement in alveolar structure and infiltration of immune cells leading to thickening of alveolar wall (➛), indicating structural damage compared to lung tissues of normal mice (Normal). Moreover, Tumor showed higher expression of cleaved PARP (brown) in the lung tissue. 25 mg/kg ART-treated tumor-bearing (T+25) indicated that the aberrations were rectified. Similarly, liver tissue from the Tumor, showed hepatocyte ballooning (➛) around the central vein (CV) indicating apoptotic bodies along with infiltration of immune cells. Liver tissue from the Tumor also has high expression of cleaved PARP (brown) compared to the Normal. Such structural changes and heightened cleaved PARP expression was not observed in T+25 liver tissue. Kidney tissue from the Tumor showed disrupted glomerular (G) organization, indicating severe cellular death in these structures. Similar to the other tissues, kidney from Tumor showed a higher expression of cleaved PARP in the glomerulus and surrounding tubules compared to the Normal. However, these changes were rectified in T+25 ART-treated kidneys. Bar: 20µm. Each experiment was repeated three times and representative images are shown.

Supplementary Figure 2

(A)

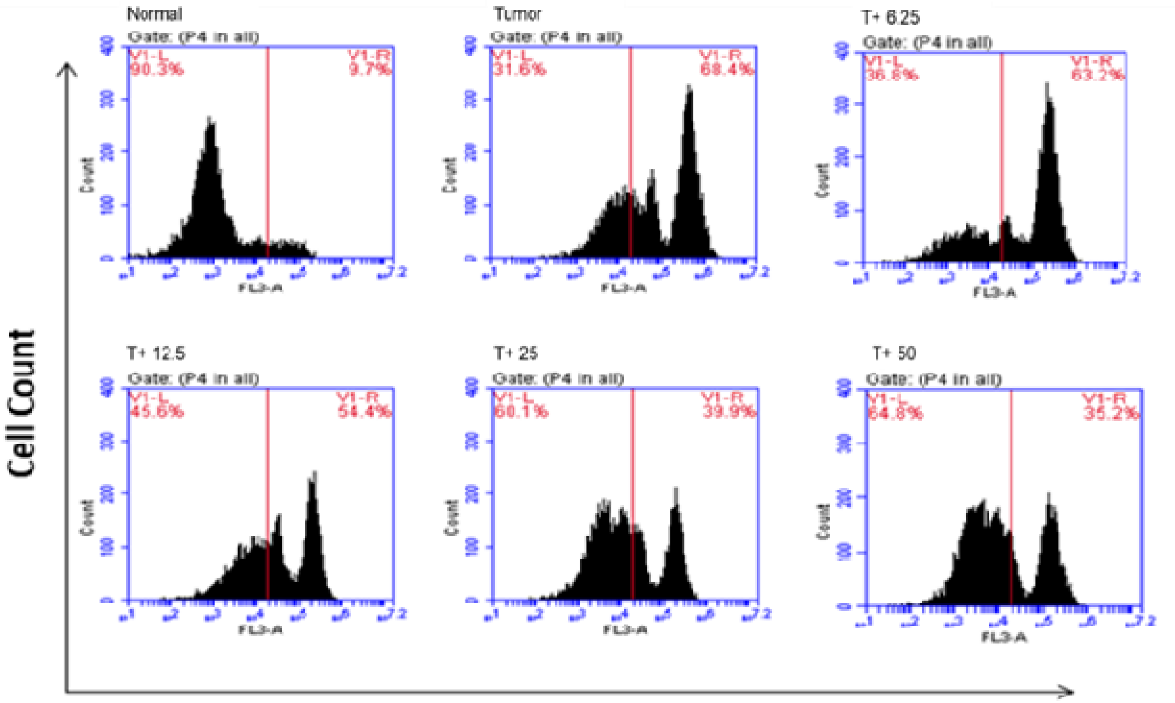

(B)

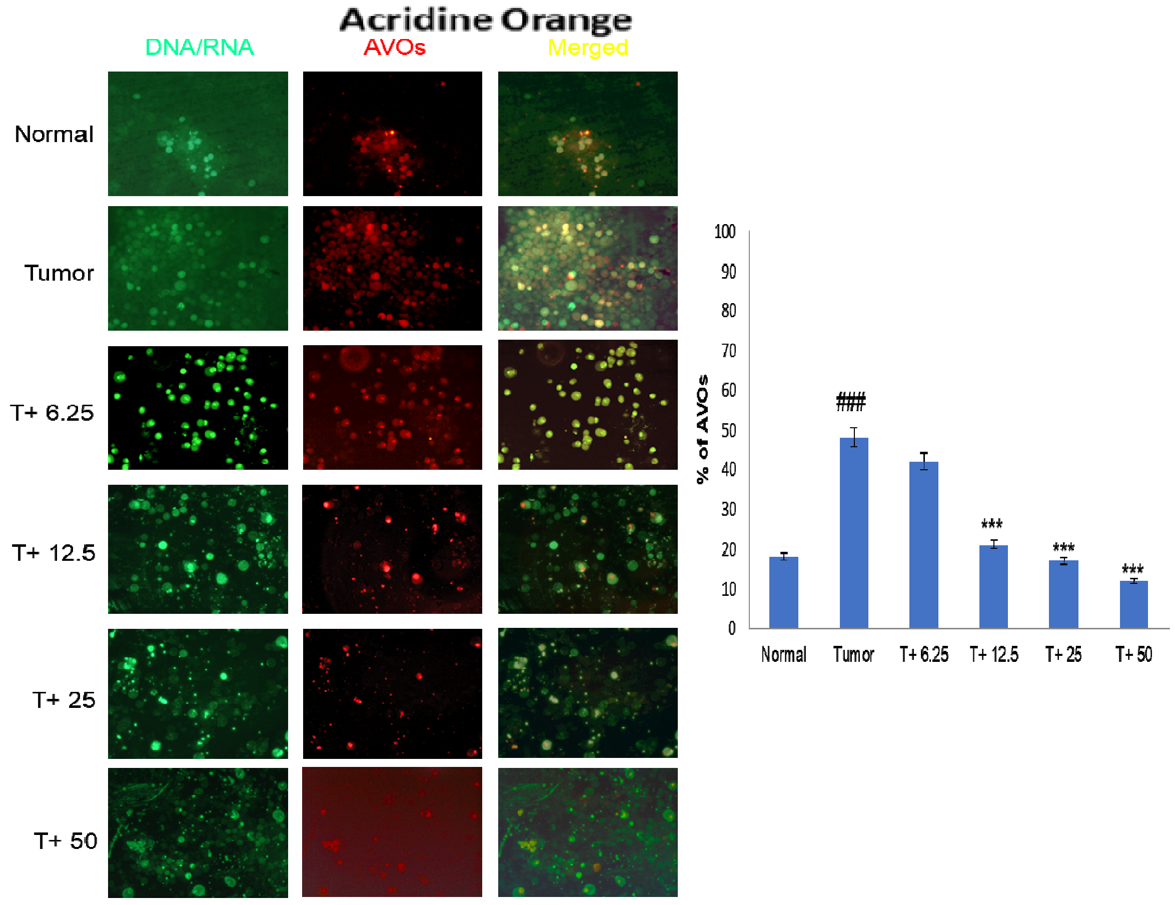

Supplementary Figure 2. ART treatment in tumor bearing mice decreases the percentage of autophagic cells. **A**. Cells were analyzed in a flow cytometer, from normal mammary fat pad tissue of normal mice (Normal) and, untreated (Tumor) and ART treated (T+6.25, T+12.5, T+25 and T+50 mg/kg bodyweight) tumor-bearing mice. It was observed that there was a 7 fold increase in the percentage of autophagic cells (right quadrant; V1-R) between the Tumor and Normal. Upon ART treatment there was a dose-dependent decrease in the percentage of autophagic cells. **B**. Cells isolated from Normal, Tumor and ART treated tumor-bearing mice at different doses (T+6.25, T+12.5, T+25 and T+50) were observed under a fluorescent microscope at 10x magnification and it revealed acidic vesicular organelles (AVOs; Red) increased in Tumor samples ($p<0.001$) compared to the Normal. Upon ART treatment there was a significant dose-dependent reduction AVOs compared to the Tumor. $^{###}p<0.001$, compared to the Normal; ***$p<0.001$; compared to the Tumor; n=5.

Supplementary Table 1. Biochemical parameters of normal mice treated with ART for 3 weeks. Serum levels of ALT, AST, creatinine and urea were assessed. Normal mice were treated with the vehicle only. No significant changes were noted in mice treated with varying doses of ART for 21 days in comparison to untreated normal mice (n=5). Each analysis was performed three times.

**ART (mg/kg body wt)**	**ALT (U/l)**	**AST (U/l)**	**Creatinine (mg/dl)**	**Urea (mg/dl)**
Normal	27.4±3.9	30.6±3.3	0.52±0.09	29.1±2.3
6.25	28.1±2.6	29.8±4.6	0.48±0.21	31.6±1.9
12.5	29.3±3.1	31.4±2.6	0.68±0.12	28.7±2.3
25	29.5±2.1	28.5±2.8	0.49±0.3	30.1±2.6
50	28.8±3.2	30.3±1.9	0.62±0.38	28.9±2.1
