# Supplementary figures and images for: CLEC12A sensitizes differentially responsive breast cancer cells to the anti-cancer effects of artemisinin by repressing autophagy and inflammation
Source: Front Oncol. 2023 Dec 8;13:1242432. doi: 10.3389/fonc.2023.1242432 (PMC10748408; doi:10.3389/fonc.2023.1242432)

**A**

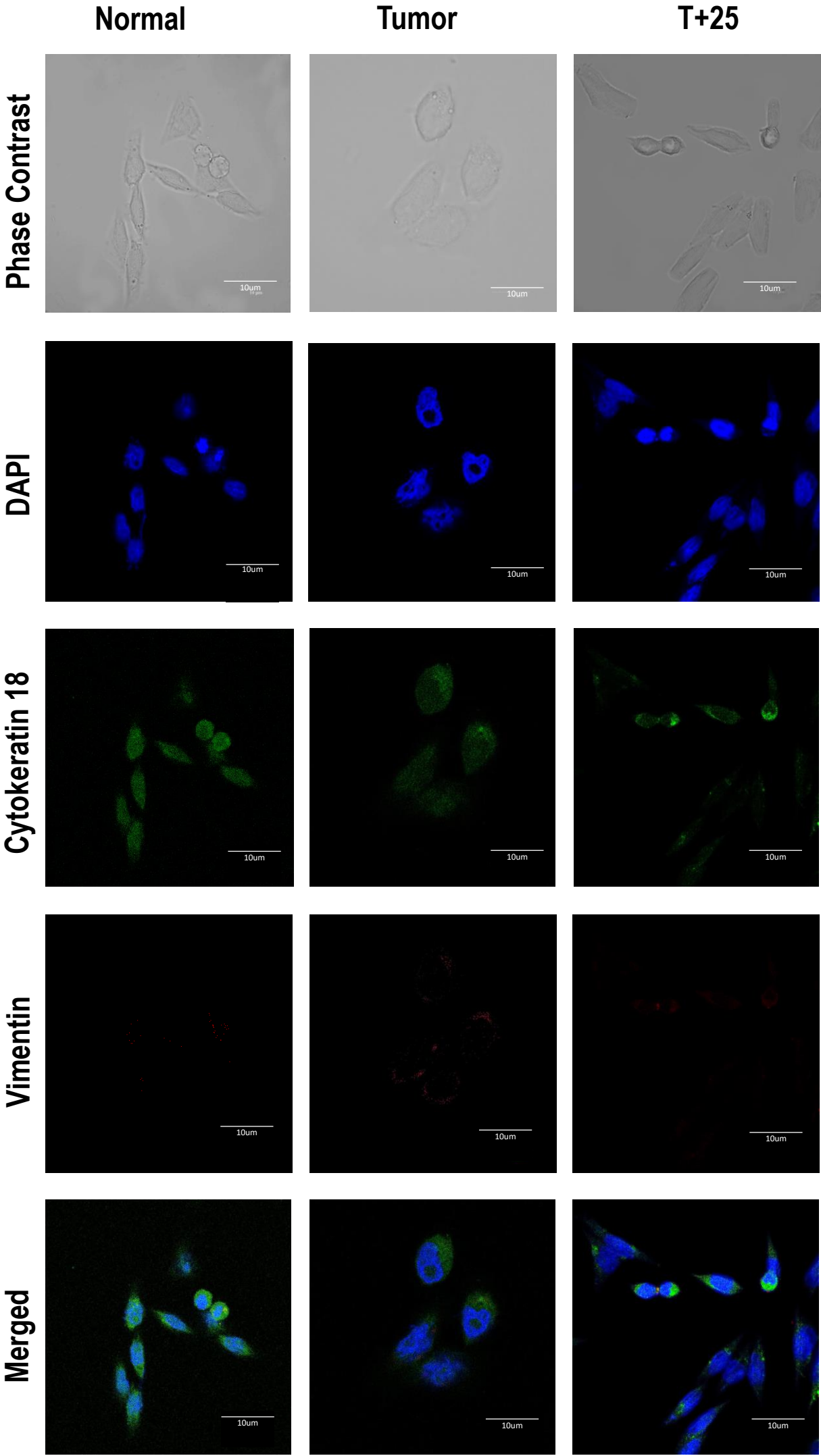

**B**

Normal

Tumor

T+25

H&amp;E

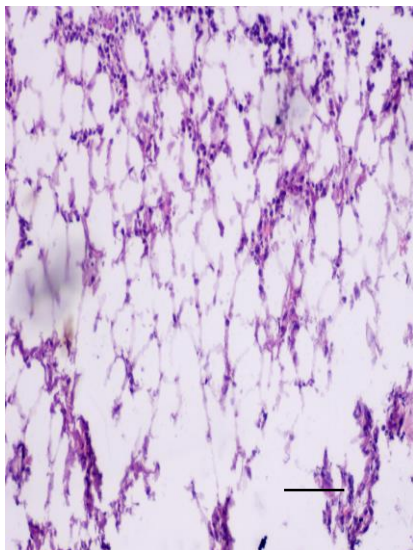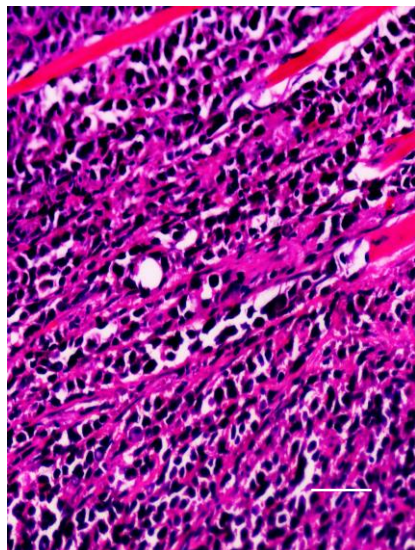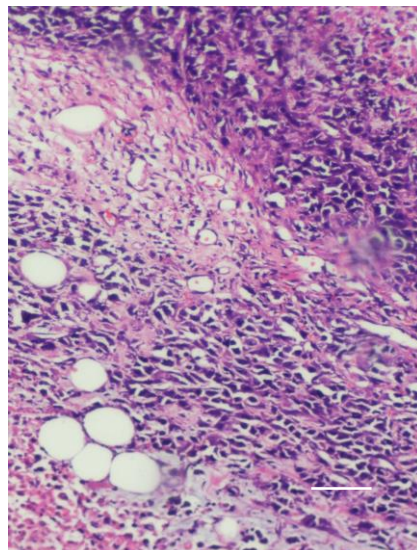

DAPI

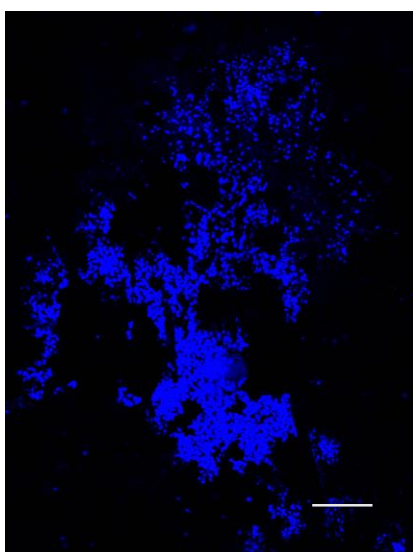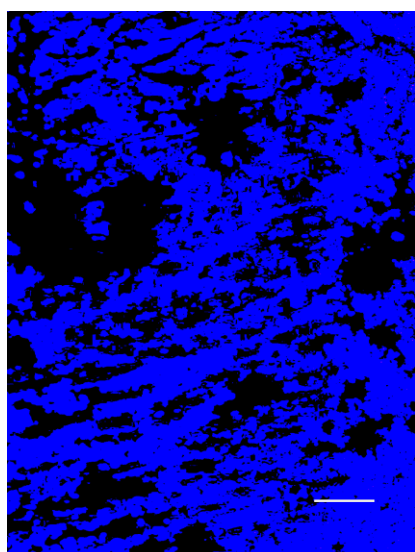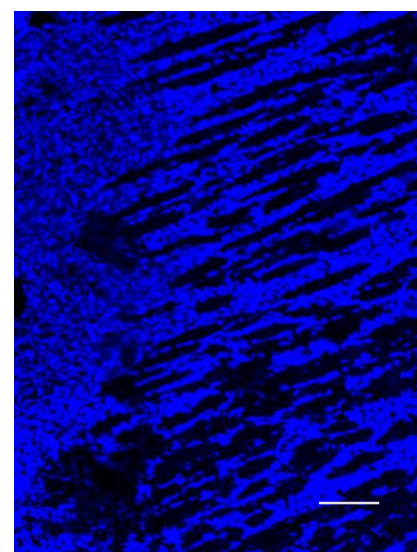

TUNEL

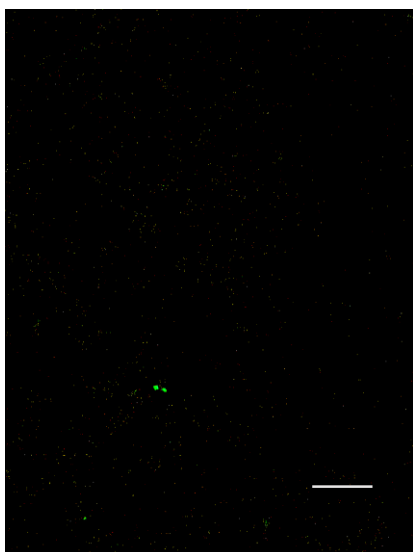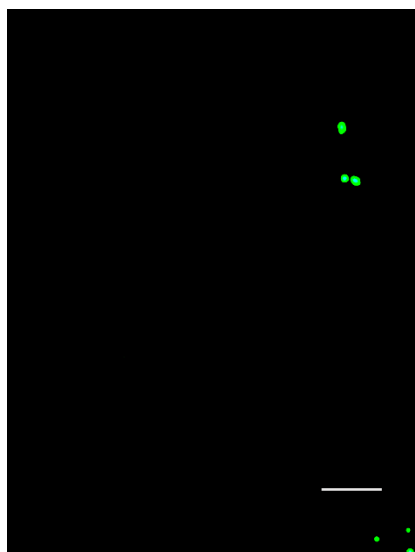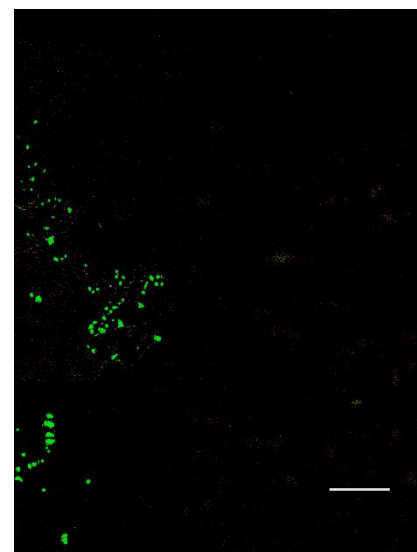

Merged

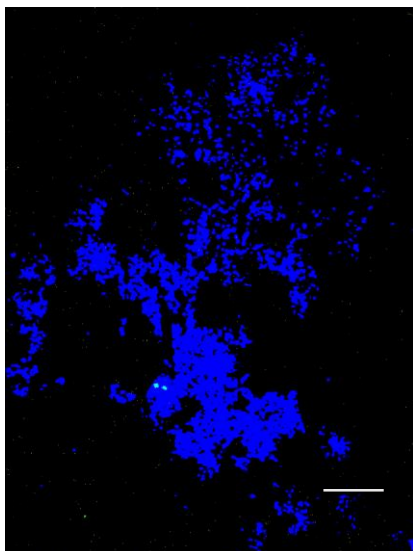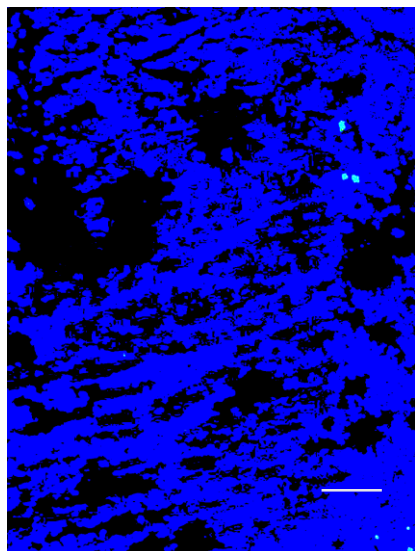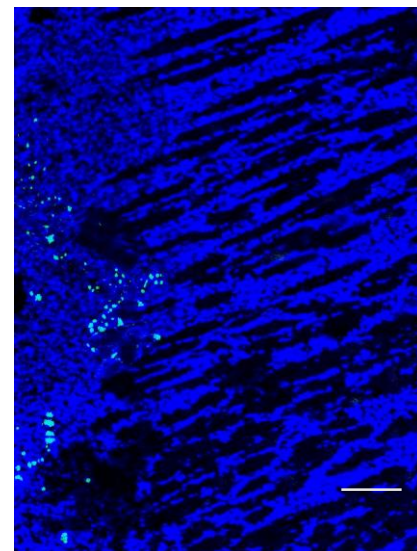

Supplement: Supplementary file 2 [file DataSheet_2.pdf]
